# Sex differences in renal cell carcinoma: a single-cell analysis reveals exhausted CD8^+^ T-cells highly infiltrated in males

**DOI:** 10.1186/s13293-023-00540-9

**Published:** 2023-09-15

**Authors:** Kang Ning, Yulu Peng, Yue Jiang, Zhen Li, Xin Luo, Lede Lin, Minhua Deng, Yi Wu, Tingxuan Huang, Yixin Huang, Ye Xie, Xiaofeng Yang, Manhuai Zhang, Longbin Xiong, Xiangpeng Zou, Zhaohui Zhou, Fangjian Zhou, Pei Dong, Chunping Yu, Zhiling Zhang

**Affiliations:** 1 Department of Urology, Sun Yat-Sen University Cancer Center, Guangzhou, China; 2 State Key Laboratory of Oncology in Southern China, Collaborative Innovation Center for Cancer Medicine, Guangzhou, China; 3 Department of Colorectal Surgery, Guangdong Provincial Key Laboratory of Colorectal and Pelvic Floor Disease, The Sixth Affiliated Hospital, Sun Yat-Sen University, Guangzhou, China; 4 Guangdong Research Institute of Gastroenterology, The Sixth Affiliated Hospital, Sun Yat-Sen University, Guangzhou, China; 5 Department of Urology, Hunan Provincial People’s Hospital, The First Affiliated Hospital of Hunan Normal University, Changsha, China; 6 Department of Urology, Institute of Urology (Laboratory of Reconstructive Urology), West China Hospital, Sichuan University, Chengdu, China; 7 State Key Laboratory of Ophthalmology, Zhongshan Ophthalmic Center, Sun Yat-Sen University, Guangzhou, China; 8 Zhongshan School of Medicine, Sun Yat-Sen University, Guangzhou, China; 9 Guangdong Key Laboratory of Urology, Department of Urology, Minimally Invasive Surgery Center, Institute of Urology, The First Affiliated Hospital of Guangzhou Medical University, Guangzhou, China

**Keywords:** Sex bias, Renal cell carcinoma, Single-cell transcriptomics, Immunotherapy, T-cell, Exhaustion, Androgen

## Abstract

**Background:**

Although sex bias has been reported in the development and progression of renal cell carcinoma (RCC), the underlying mechanisms remain enigmatic. Here, we investigated the sex differences in the tumor microenvironment (TME) of RCC and explored a promising combination drug regimen to enhance the efficacy of immunotherapy.

**Methods:**

Single-cell RNA sequencing (scRNA-seq) data from four published datasets were analyzed to investigate the sex differences in RCC patients, and tumor tissues were collected to validate the sex differences using multiplex immunofluorescence (MxIF) and flow cytometry (FCM). The function of the androgen–androgen receptor axis in sex differences was explored in vivo and in vitro experiments.

**Results:**

Our analysis of scRNA-seq data from 220,156 cells, as well as MxIF and FCM assays, revealed that CD8^+^ T-cells infiltrated highly in the TME of male RCC, but were mostly in an exhausted and dysfunctional state. In vitro and in vivo experiments indicated that the dysfunction and exhaustion of CD8^+^ T-cells in male TME were induced by androgen. Clinically, higher serum androgen was significantly associated with a worse prognosis in male RCC patients receiving immunotherapy. Androgen receptor inhibitors could activate tumor-infiltrating CD8^+^ T-cells and enhance the efficacy of immunotherapy of RCC in vivo.

**Conclusions:**

Our study delineated the difference in TME between male and female patients with RCC, and demonstrated that the androgen–androgen receptor axis plays an important role in immunosuppression in male RCC. Our findings suggest that androgen receptor inhibitors in combination with immunotherapy may be a promising treatment option for male RCC patients.

**Supplementary Information:**

The online version contains supplementary material available at 10.1186/s13293-023-00540-9.

## Highlights

1. Male renal cell carcinoma is characterized by a heightened degree of malignant cell malignancy, with classical tumorigenic pathways, including EMT, angiogenesis, and TGF-β, highly enriched in the malignant cells.
2. No significant difference in the subsets of infiltrating myeloid cells, indicating that myeloid cells may not play a significant role in the gender bias of renal cell carcinoma.
3. We observed a higher infiltration of CD8^+^ T-cells in male renal cell carcinoma, most of which were in an exhausted state.
4. The androgen–androgen receptor axis leads to CD8^+^ T-cell exhaustion in male renal cell carcinoma, resulting in the compromised anti-tumor function of these cells.
5. Androgen receptor inhibitors may hold the potential to alleviate the exhaustion of CD8^+^ T-cells, paving the way for a promising combination therapy with immune checkpoint inhibitors for immunotherapy.

**Supplementary Information:**

The online version contains supplementary material available at 10.1186/s13293-023-00540-9.

## Background

Renal cell carcinoma (RCC) is a prevalent cancer of the urinary system that poses a significant threat to public health, with an annual global incidence of approximately 2% over the past two decades [1, 2]. However, emerging evidence suggests the existence of sex bias in the development and progression of RCC. Population-based studies have revealed that the incidence ratio of RCC in males to females is 2:1, regardless of age, time period, or geographic location [3]. Furthermore, compared to their female counterparts, male RCC patients frequently exhibit larger tumors, higher stage and grade, and worse prognosis [4–6]. These findings underscore the importance of considering sex as a biological variable in the diagnosis, treatment, and management of RCC, and highlight the need for further investigation into the underlying mechanisms driving sex-specific differences in RCC.

Immune checkpoint inhibitors (ICIs) have emerged as the standard first-line treatment and could improve prognosis in RCC [7, 8]. However, there is ongoing debate about whether the benefits of immunotherapy for RCC differ between males and females. Notably, a meta-analysis of studies revealed that male patients with metastatic RCC experienced a greater benefit from ICIs than their female counterparts [9].The role of sex-hormone modulation has been described in the PD-1/PD-L1 pathway, and the hormonal effects on the PD-1 pathway are important in mediating autoimmunity [10, 11]. However, based on data from the International Metastatic Renal Cell Carcinoma Database Consortium (IMDC), Graham et al. found no evidence of sex-dependent differences in the magnitude of immunotherapy benefit for RCC patients [12]. Thus, more in-depth research on sex bias in the occurrence, development, and treatment of RCC is necessary.

Sex chromosomes and hormones were reported to be associated with sex bias in RCC. Dunford et al. discovered that a subset of genes on the X-chromosome could evade X-inactivation, providing females with some level of protection from complete functional loss due to a single mutation [13]. Prior studies have also shown that estrogen can increase apoptosis in RCC, while androgen and the androgen receptor (AR) can promote various aspects of tumor progression, including stemness, angiogenesis, proliferation, migration, and invasion of RCC [14–21]. Overall, these findings suggest that the sex-specific effects of sex hormones and sex chromosome genes may play a crucial role in the development and progression of RCC, and further investigation in this area is warranted.

Tumor microenvironment (TME) is a complex ecosystem composed of several cell types, including immune cells, tumor cells, and extracellular matrix, which plays a critical role in tumor development and progression [22]. Although males and females have been confirmed to display distinct innate and adaptive immune responses [23], previous studies have reported conflicting findings regarding the influence of gender on TME in different types of cancer. Conforti et al. observed that the TME of female lung cancer patients was characterized by significantly greater T-cell dysfunction status and higher expression of inhibitory immune checkpoint molecules [24]. Comparatively, other studies reported that androgen-mediated promotion of CD8^+^ T-cell dysfunction resulted in a suppressive immunological state of male TME in bladder and liver cancer [25–27]. While several studies have investigated sex bias in RCC, most of them have focused on tumor cells, and the role of immune cells in TME has not been fully explored [14–21]. Given the importance of TME in cancer progression, a comprehensive understanding of sex differences in RCC TME is crucial to develop sex-specific therapeutic strategies.

In this study, published single-cell RNA sequencing (scRNA-seq) datasets of primary RCC were utilized to analyze the sex differences in the TME and their effects on sex bias and immunotherapy outcomes. Multiplex immunofluorescence (MxIF), immunohistochemistry (IHC), and flow cytometry (FCM) were performed to confirm the observations, and the efficacy of a combination regimen, ICIs plus androgen receptor inhibitors (ARi), based on sex differences was assessed in vivo.

## Methods

### Study approval

This study was performed in accordance with the ethical standards of the Helsinki Declaration and the ethical guidelines for Medical and Health Research Involving Human Subjects and approved by the Ethics Review Board of Sun Yat-sen University Cancer Center (SYSUCC). Human primary RCC tissues for FCM were obtained from the operating room of SYSUCC during nephrectomy and written informed consent was obtained from all patients. All serum and pathological specimens from RCC patients were collected from the SYSUCC Bio-bank. Mouse experiments were performed in a specific pathogen-free environment at the animal laboratory of the SYSUCC according to institutional guidelines, and all animal experimental protocols were approved and reviewed by the Ethics Review Committee for Animal Experimentation of SYSUCC.

### Experimental design

Publicly available single-cell RNA-seq datasets were downloaded from the Genotypes and phenotypes (phs002065, phs002252), Sequence Read Archive (SRP308561), and European Genome-phenome Archive (EGAS00001002325) [8, 28–30]. Heterogeneity among different data sets was controlled by strictly uniform sample inclusion criteria and analysis procedures. Sex-related genes (XIST, RPS4Y1) were used to identify sex groups in each sample. To validate findings in single-cell analysis, sixty primary RCC samples were collected for MxIF, and ten for FCM. Serum androgen and IHC from 44 RCC patients and CD8^+^ T-cell cytotoxicity assay were used to testify the association between serum androgen and CD8^+^ T-cells exhaustion in RCC CD8^+^ T-cell. The role of androgen and ARi has been demonstrated in vivo by xenograft mouse models and IHC. For further details regarding the materials and methods, please refer to Additional file 1.

## Results

### Clinical data analysis and single-cell transcriptome atlas of primary RCC

**Fig. 1:**
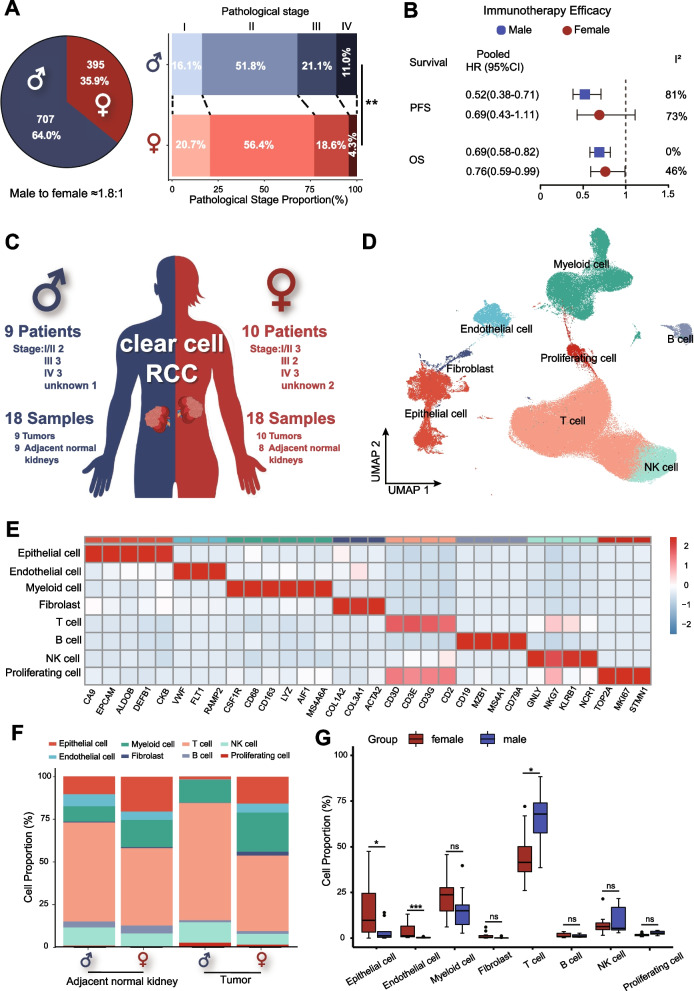
scRNA-seq profiling of TME of male and female RCC patients. A Sex difference of incidence and clinical stages of 1102 RCC patients who underwent nephrectomy from 1999 to 2020 at SYSUCC. **B** Meta-analysis of sex-specific pooled hazard ratios in various immunotherapy RCTs. **C** A schematic representation of scRNA-seq profiling of tumors and adjacent normal kidneys in male and female patients with RCC. **D** The UMAP plot shows the annotation and color codes for different cell types in tumors and adjacent normal kidney ecosystems. **E** Heatmap showing the expression of marker genes in the indicated cell types. **F**, **G** Histograms and boxplots illustrating the percentage of cell types in different groups. HR: hazard ratio; OS: overall survival; PFS: progression-free survival; RCC: renal cell carcinoma; RCTs: randomized controlled trials; scRNA-seq: single-cell RNA sequencing; SYSUCC: Sun Yat-sen University Cancer Center; UMAP: uniform manifold approximation and projection

After analysis of 1102 RCC patients receiving radical nephrectomy at the Sun Yat-sen University Cancer Center (SYSUCC), we found the incidence ratio of male to female RCC was about 1.8:1, and the pathological stage of male RCC patients was significantly higher than females (Fig. 1A). After a meta-analysis of five randomized controlled trials of immunotherapy in RCC, we found that male RCC patients had increased benefits from immunotherapy, especially regarding progression-free survival (PFS) (Fig. 1B and Additional file 2: Fig. S1A).

We summarized the currently published scRNA-seq of primary RCC from four studies [8, 28–30]. After screening, 18 samples (9 tumor and adjacent normal kidney samples) from 9 males and 18 samples (10 tumor samples and 8 adjacent normal kidney samples) from 10 females were included in this study (Additional file 2: Fig. S1B and Additional file 3: Table S1B). The female and male patients included in this study had relatively similar clinical stages (Fig. 1C). The expression of sex-related genes (XIST, RPS4Y1) was used to identify the gender of each patient in this study (Additional file 2: Fig. S1C).

After quality control and removal of the batch effect between samples, 220,156 single cells were clustered and identified into 8 major cell lineages using the uniform manifold approximation and projection (UMAP) method (Fig. 1D and Additional file 2: Fig. S2A, B): epithelial cell, endothelial cell, myeloid cell, fibroblast, T cell, B cell, natural killer cell (NK cell), and proliferating cell. The heatmap of marker genes showed the homogeneity of each major cell lineage (Fig. 1E). Interestingly, compared to the TME of females, a higher infiltration level of T-cells was found in male TME (p < 0.05, Fig. 1G). Each tumor had a relatively uniform cell lineage composition (Additional file 2: Fig. S2C), while the adjacent normal kidney differed widely (Additional file 2: Fig. S2D).

### Tumor cells from males were more malignant than females

**Fig. 2:**
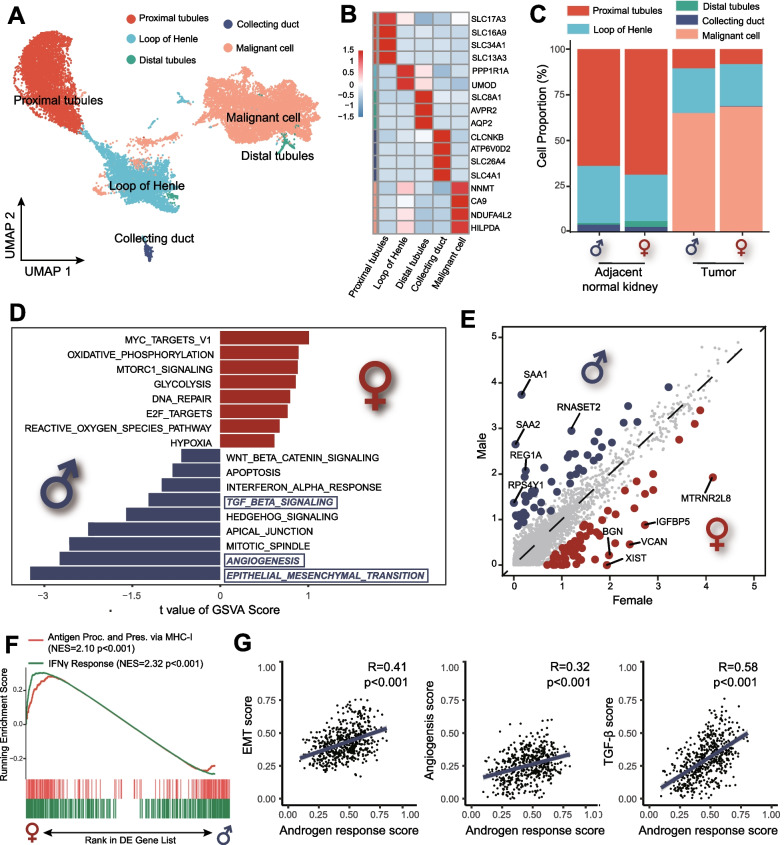
Identification and characterization of epithelial cells and malignant cells in males and females. **A** UMAP plot representing the subtypes of epithelial cells and malignant cells from male and female samples. **B** Heatmap showing the expression of marker genes in the epithelial cells and malignant cells. **C** Histogram showing the percentage of epithelial cells and malignant cells in samples and groups. **D** GSVA analysis showing the enrichment of specific pathways in malignant cells based on the HALLMARK gene set. **E** The volcano plot showing DEGs between male (blue dots) and female (red dots) malignant cells. **F** GSEA of hallmark interferon-γ response and gene ontology antigen presentation and processing via MHC class I signatures in malignant cells between male and female. **G** The correlation analysis between androgen response score and EMT, angiogenesis and TGF-β score in male malignant cells. DEGs: differential genes expression; EMT: epithelial to mesenchymal transition; GSEA: gene set enrichment analysis; GSVA: gene set variation analysis; TGF-β: transforming growth factor-β; UMAP: uniform manifold approximation and projection

The above-identified epithelial cells were re-clustered and identified into five cell types: proximal tubules, the loop of Henle, distal tubules, collecting duct, and malignant cells (Fig. 2A, B). The histogram illustrates that the malignant cells were concentrated in the tumor samples (Fig. 2C). The gene set variation analysis (GSVA) based on HALLMARK gene sets indicated a highly activated state of epithelial–mesenchymal transition (EMT), angiogenesis, and transforming growth factor-β (TGF-β) pathways in male malignant cells (Fig. 2D).

We further examined the differentially expressed genes (DEGs) in malignant cells between males and females (Additional file 3: Table S2A). The expression of SAA1/2, as non-sex-related genes, was significantly higher in male malignant cells (Fig. 2E). The upregulation of SAA1/2 could increase the invasive potential of tumor cells in RCC and lead to massive T-cell infiltration [31, 32], which might explain the sex bias of malignant cells and T-cells infiltration.

Next, we examined the immune escape status of malignant. Compared with females, male malignant cells downregulated gene sets associated with interferon-γ response and antigen processing on major histocompatibility complex (MHC) class I, indicating the immune escape propensity of male tumors (Fig. 2F). Previous studies reported that androgen could promote tumor stemness and angiogenesis and enhance the proliferation, migration, and invasion of RCC [14]. In single-sample gene set enrichment analysis (ssGSEA), we also found that androgen response scores were highly correlated with EMT scores (R = 0.41, *p* < 0.001), angiogenesis score (R = 0.32, *p* < 0.001), and TGF-β scores (R = 0.58, *p* < 0.001) in male malignant cells (Fig. 2G).

### Identification and characterization of myeloid cells in different gender

All myeloid cells were extracted and identified into 7 subtypes: type 1 conventional dendritic cells (cDC1), type 2 conventional dendritic cells (cDC2), tumor-associated macrophages (TAM), resident-tissue macrophages (RTM), CD14 monocytes, CD16 monocytes, and DEGs in each cluster were also summarized (Additional file 3: Fig. S3A–C). There was a significantly higher proportion of TAM in the TME compared to the adjacent normal kidney in both males and females, while the proportion of CD16 monocytes was significantly lower (Additional file 3: Fig. S3D, E). However, there was no significant difference in each subtype of myeloid cells between the TME of males and females.

GSVA indicated that female infiltrating macrophages had high activation of several pro-inflammatory signaling pathways in either adjacent normal kidneys (Additional file 2: Fig. S4A) or tumors (Additional file 2: Fig. S4B), including TH2 activation, lymphocyte activation, complement activation, cytokine signaling, and antigen presentation. Then, we separately analyzed the trajectories of macrophages in males and females to investigate their transition states. However, no significant difference was observed in the distribution of macrophages along the pseudotime between males and females in either the TME or adjacent normal kidneys (Additional file 2: Fig. S4C, D).

### T/NK cell clustering reveals highly infiltrating and exhausted CD8^+^ T-cells in male TME of RCC

**Fig. 3:**
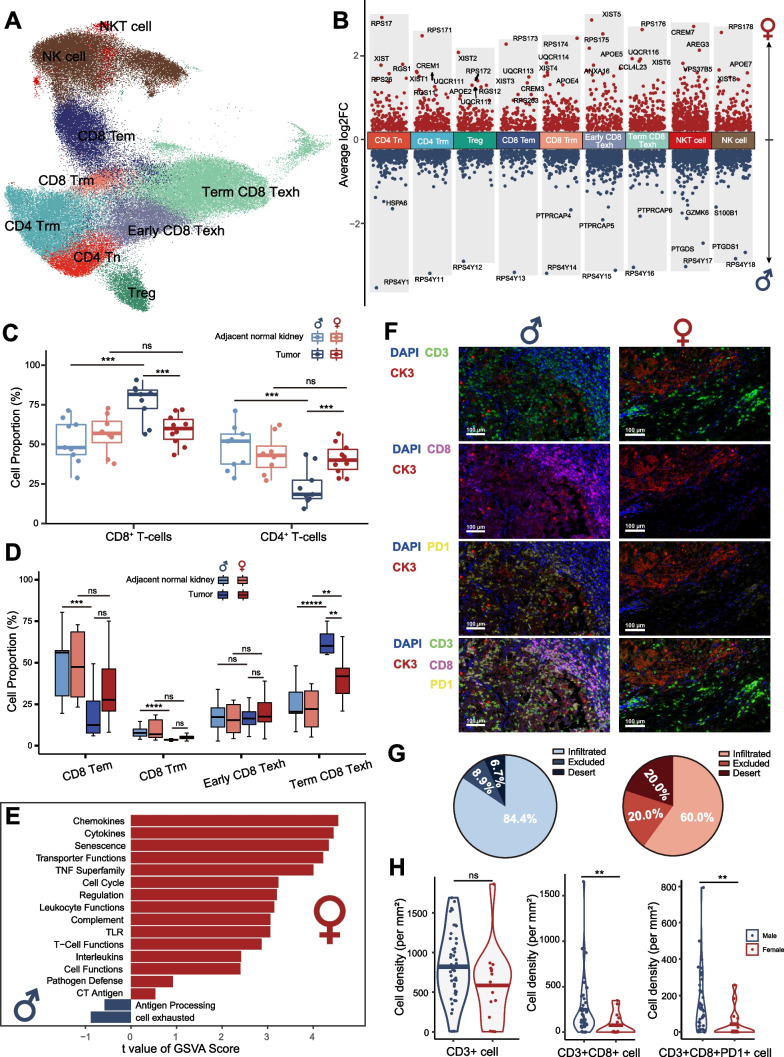
Characteristics of infiltrating T/NK cells in males and females. **A** The UMAP plot showing different T/NK cell subtypes, colored and labeled by cell type. **B** The volcano plot showing DEGs between males (blue dots) and females (red dots) in different T-cells subtypes. **C** Boxplots illustrating the percentage of infiltrating CD4^+^ and CD8^+^ T-cells in the tumor and adjacent normal kidneys of males and females. **D** Boxplots illustrating the percentage of infiltrating CD8^+^ T-cell subtypes in tumor and adjacent normal kidneys of males and females. **E** Differentially enriched pathways were scored per cell by GSVA in tumor-infiltrating CD8^+^ T-cells between males and females. **F** MxIF images of male and female tumors demonstrating tumor-infiltrating CD3^+^CD8^+^PD1^+^ T-cells. **G** The pie charts showing the percentage of CD8^+^ T-cell infiltration types. **H** Violin plots demonstrating CD3^+^, CD3^+^ CD8^+^, CD3^+^ CD8^+^ PD1^+^ infiltration levels in MxIF of tumors from males (*n* = 45) and females (*n* = 15). DEGs: differential gene expression; GSVA: gene set variation analysis; MxIF: multiplex immunofluorescence; UMAP: uniform manifold approximation and projection

We performed unsupervised clustering of T and NK cells and identified 9 subtypes: CD4^+^ naïve T-cells (CD4^+^ Tn), tissue-resident memory CD4^+^ T-cells (CD4^+^ Trm), regulatory T-cells (Treg), effector memory CD8^+^ T-cells (CD8^+^ Tem), tissue-resident memory CD8^+^ T-cells (CD8^+^ Trm), early exhausted CD8^+^ T-cells (early CD8^+^ Texh), terminal exhausted CD8^+^ T-cells (term CD8^+^ Texh), natural killer T-cells (NKT cell), and NK cells (Fig. 3A, Additional file 2: Fig. S5A, B). DEGs in each T-cell subtype between males and females showed that the sex-related genes were significantly different in each T-cell subcluster (Additional file 3: Table S3A and Additional file 2: Fig. S3B).

There was a higher infiltration of CD8^+^ T-cells (CD8^+^ Tem, CD8^+^ Trm, early CD8^+^ Texh, term CD8^+^ Texh) in male TME as opposed to female TME, while CD4^+^ T-cells (CD4^+^ Tn, CD4^+^ Trm, Treg) were less abundant (Fig. 3C and D). Interestingly, although female and male TME both had a high level of term CD8^+^ Texh compared to adjacent normal kidneys, male TME had a significantly higher level of term CD8^+^ Texh infiltration (Fig. 3D). However, there was no significant difference in all CD4^+^ T-cell subtypes between male and female TME (Additional file 2: Fig. S5C).

Then, we performed GSVA in CD8^+^ T-cells in both adjacent normal kidneys and the tumor. Cell-toxicity or cell-exhaustion signature pathways were all highly enriched in female adjacent normal kidneys, which indicated a quite active renewal of CD8^+^ T-cells in females (Additional file 2: Fig. S5D). In the tumor cell-toxicity signature pathways, including chemokines, cytokines, and transporter functions, were highly enriched in females, whereas cell-exhaustion signature pathways were highly enriched in males in tumor-infiltrating CD8^+^ T-cells (Fig. 3E).

To further validate the highly infiltrating and exhausted CD8^+^ T-cells in male TME of RCC, we performed MxIF in high-quality tumor samples from RCC patients who had undergone radical nephrectomy (n = 60). We found higher tumor-infiltrating CD3^+^CD8^+^ T-cells and CD3^+^CD8^+^PD1^+^ T-cells in males compared to females (Fig. 3F). The majority of RCC (84.4%) in males were the infiltrative type, while there were more excluded (20.0%) and desert (20.0%) types in female RCC (Fig. 3G). The average density of CD3^+^CD8^+^ T-cells (p < 0.001) and CD3^+^CD8^+^PD1^+^ T-cells (p < 0.001) was significantly higher in male RCC compared with female RCC (Fig. 3G). After regrouping the previously published MxIF data of RCC [33], we confirmed the high-infiltration level of CD3^+^CD8^+^ T-cells in the male TME (Additional file 2: Fig. S5E).

### Exhaustion of CD8^+^ T-cells in RCC by trajectory analysis

**Fig. 4:**
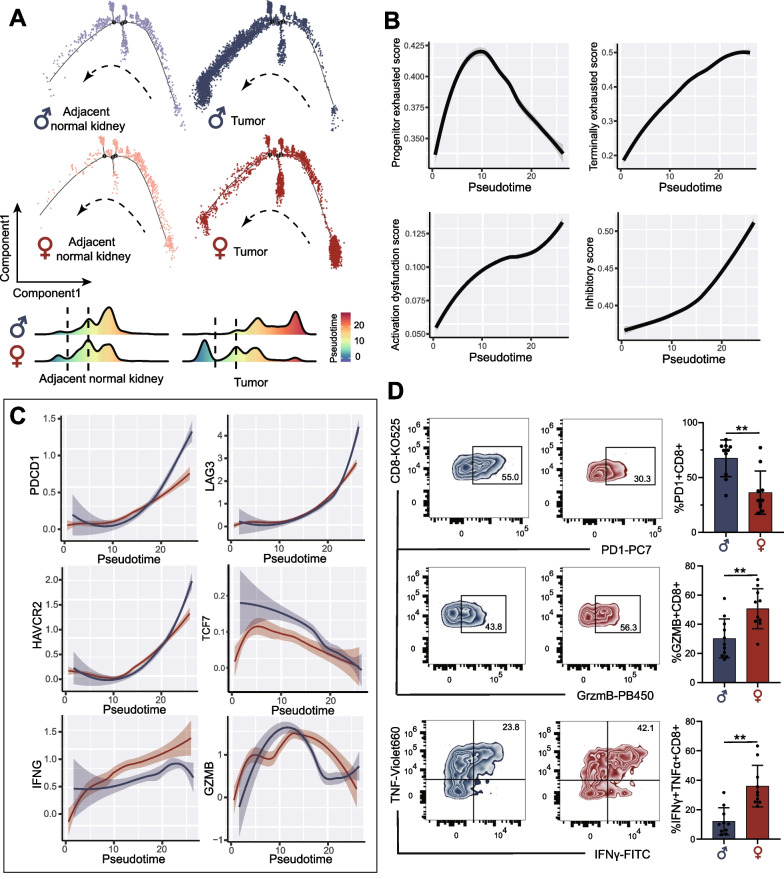
Analysis of infiltrating CD8^+^ T-cell transition states in male and female samples. **A** Pseudotime-ordered analysis and density-distribution map of infiltrating CD8^+^ T-cells in tumors and adjacent normal kidneys of males and females. **B** Two-dimensional plots showing the change of expression scores for genes related to T-cell exhaustion and dysfunction along with the pseudotime. **C** Two-dimensional plots showing the dynamic expression of exhaustion and cytotoxicity genes during the CD8^+^ T-cell transitions along the pseudotime in male (blue) and female (red) samples. **D** The exhausted status and cytotoxic function of tumor-infiltrating CD8^+^ T-cells assessed by flow cytometry in male (*n* = 10) and female tumor (*n* = 10) samples

We explored the dynamic immune states and gene expression of CD8^+^ T-cells in tumors and adjacent normal kidneys by inferring the state trajectories using Monocle. Male and female tumors both showed more CD8^+^ T-cells in terminal pseudotime than adjacent normal kidneys. However, unlike in females, CD8^+^ T-cells in male RCC peaked at the end-stage of pseudotime (Fig. 4A). Along the pseudotime, the terminally exhausted score, activation dysfunction score, and inhibitory score increased continuously, while the progenitor exhausted score peaked in the middle of pseudotime and then decreased (Fig. 4B). The above findings suggested that the distribution of CD8^+^ T-cells in pseudotime reflected the level of exhaustion, and tumor-infiltrating CD8^+^ T-cells in males were mostly exhausted.

Next, we investigated the exhaustion and cytotoxicity of gene changes along with pseudotime in tumor-infiltrating CD8^+^ T-cells (Fig. 4C). The expression level of exhaustion genes, such as *PDCD1*, *LAG3,* and *HAVCR2*, increased along the pseudotime, while males showed a higher level of expression than females. Along the pseudotime, *IFNG* increased in males, but at a lesser rate than females, and declined at the end of the period. GrzmB increased to the peak and then decreased along the pseudotime, but the decrease in males was greater than that in females.

High-dimensional flow cytometry (FCM) analysis of available RCC samples (males: 10, females: 10) was performed to validate the protein-level expression of inferred receptors and ligands on CD8^+^ T-cells (expressing CD45^+^CD3^+^CD8^+^, Additional file 2: Fig. S6A). As shown in Fig. 4D, more exhausted CD8^+^ T-cells (expressing CD8^+^ PD1^+^, p < 0.001) and fewer cytotoxic CD8^+^ T-cells (expressing CD8^+^ GrzmB^+^, p < 0.001) infiltrated in male TME. Besides, tumor-infiltrating CD8^+^ T-cells in males produced less IFNγ and TNF than in females. As a result, although a higher infiltration level of CD8^+^ T-cells was found in male TME, those CD8^+^ T-cells were mainly exhausted and dysfunctional.

### Androgen was involved in the dysfunction and exhaustion of CD8^+^ T-cells in male RCC

**Fig. 5:**
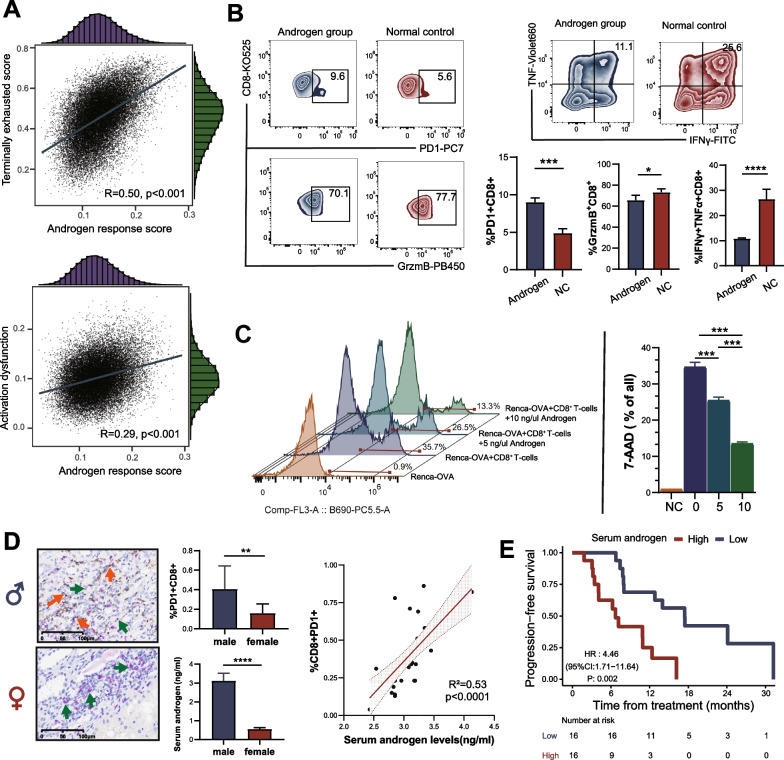
Androgen contributes to the dysfunction and exhaustion of CD8^+^ T-cells. **A** Correlation analysis between T-cells status score and androgen response score. **B** The FCM of human CD8^+^ T-cells in androgen culture. **C** CD8^+^ T-cells toxicity of OT-I mice in androgen culture. **D** IHC and ELISA analysis of 44 RCC patients receiving immunotherapy in SYSUCC (males: 32; females: 12). **E** Prognostic analysis of male RCC patients in different serum androgen after immunotherapy in SYSUCC. ELISA: enzyme-linked immunosorbent assay; FCM: flow cytometry; IHC: immunohistochemistry; HR: hazard ratio. RCC: renal cell carcinoma; SYSUCC: Sun Yat-sen University Cancer Center

ssGSEA were used to evaluate the terminally exhausted score, activation dysfunction score, and androgen response score of each CD8^+^ T cell in male tumor samples. Interestingly, the androgen response score was highly associated with the terminally exhausted score and activation dysfunction score in CD8^+^ T-cells, which indicated that androgen might contribute to the dysfunction of exhaustion of CD8^+^ T-cells in males RCC (Fig. 5A). Human CD8^+^ T-cells isolated from PBMC were cultured with androgen to further verify these observations. We found an increase in the percentage of CD8^+^PD1^+^ T-cells and a decrease in the percentage of CD8^+^ GrzmB^+^ T-cells (Fig. 5B). Moreover, androgen can significantly inhibit the secretion of IFNγ and TNFα in CD8^+^ T-cells. In CD8^+^ T-cells toxicity assays, CD8^+^ T-cells were isolated from OT-I mice and co-cultured with Renca-OVA cells. FCM showed that androgen could significantly reduce CD8^+^ T-induced Renca-OVA cell apoptosis (Fig. 5C). IHC (CD8 and PD1) and ELISA (androgen) were performed on 42 patients (32 males and 12 females) with RCC who received immunotherapy. The results showed that male RCC patients had higher androgen levels and more CD8^+^PD1^+^ T-cells. The androgen levels were significantly associated with the percentage of CD8^+^PD1^+^ T-cells (R^2^ = 0.53, *p* < 0.0001, Fig. 5D). Additionally, we found that higher serum androgen was significantly associated with a worse prognosis in male RCC patients receiving immunotherapy (Fig. 5E).

### Androgen receptor inhibitors (ARi) combined with anti-PD1 enhanced the efficacy of immunotherapy in vivo

**Fig. 6:**
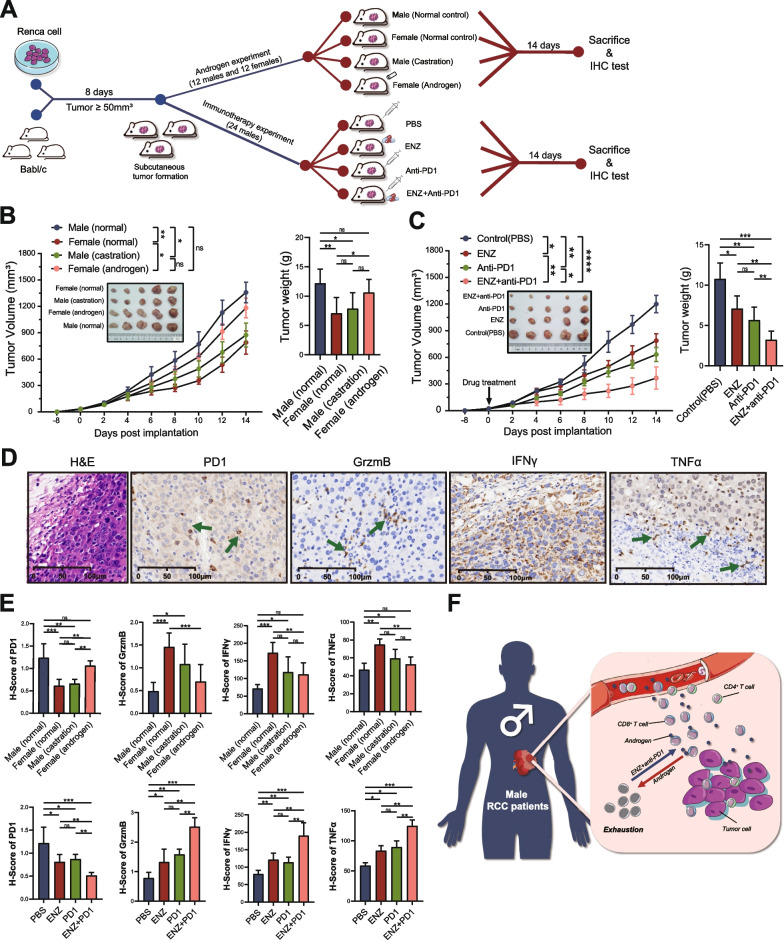
Androgen receptor inhibitors can enhance immunotherapy efficacy. **A** Flowchart of animal experiment in this study. **B**, **C** The tumor growth curves and tumor weight showing the effect of androgen and ENZ on tumor growth. **D**, **E** The expression of cytotoxicity and exhaustion markers of CD8^+^ T-cells were evaluated in mouse tumors by IHC. **F** Schematic representation showing the mechanism of androgen and androgen receptor inhibitors in RCC. ENZ: enzalutamide; IHC: immunohistochemistry; RCC: renal cell carcinoma

Considering the persistence of androgen in males, a potential therapeutic drug was required to reduce the androgen-induced dysfunction and exhaustion of CD8^+^ T-cells. We designed a series of mouse experiments to verify the effects of androgen and ARi in RCC (Fig. 6A). We found that the RCC mice grew larger after tumor formation in male mice than in female mice (Fig. 6B). However, castration in male mice inhibited tumor growth, while androgen administration in female mice did the opposite. Enzalutamide, as one of ARi, could block the effect of androgen on cells. We found that both ARi and anti-PD1 could inhibit tumor growth in male mice, but only a combination of the two demonstrated the greatest inhibition on tumor growth (Fig. 6C).

Then, we evaluated the expression of cytotoxicity and exhaustion markers of CD8^+^ T-cells using IHC in each mice group (Fig. 6D, Additional file 2: Fig. S7A, B). We found that male RCC had a higher H-score of exhaustion (PD1) and lower H-score of cytotoxicity (GrzmB, IFN-γ, and TNF-α) than females (Fig. 6E). However, castration decreased the H-score of PD1 and increased the H-score of GrzmB, IFN-γ, and TNF-α. In immunotherapy drug-sensitivity experiments, ARi combined with anti-PD1 maximized the cytotoxicity of CD8^+^ T-cells and minimized the exhaustion of CD8^+^ T-cells (Fig. 6E). As shown in the mechanism diagram, androgen led to the dysfunction and exhaustion of CD8^+^ T-cells in the TME of male RCC, while ARi activated CD8^+^ T-cells and enhanced the efficacy of immunotherapy (Fig. 6F).

## Discussion

Significant sex bias has been reported in various tumors, including RCC, whereby males tend to be affected by higher tumor incidence rates, earlier onset, more severe stage, and a worse prognosis than females in RCC [4–6]. A recent study also reported that male patients benefited more from immunotherapy in RCC [9]. As a complex micro-ecosystem, the TME plays a critical role in tumor growth, progression, metastasis, and immunotherapy response [22]. However, previous studies on sex bias in RCC mainly focused on tumor cells [14–21]. Although the female’s innate and adaptive immunity has been confirmed to be more active than the male’s [23], the exact differences in the TME between both genders remained yet to be clarified. Clarifying these differences may provide valuable insights into the mechanisms underlying sex bias in RCC and improve the development of sex-specific treatments.

In this study, we investigated the differences in the TME of RCC between male and female patients. By analyzing scRNA-seq data and validating the results using MxIF and FCS techniques, we found that male TME in RCC had a higher infiltration and exhaustion of CD8^+^ T-cells compared to females. Furthermore, classical tumor-promoted pathways such as EMT, angiogenesis, and TGF-β were highly enriched in malignant cells of male RCC. Our in vivo and in vitro experiments demonstrated that the androgen–AR axis played a critical role in inducing the exhaustion of CD8^+^ T-cells in RCC. We also observed that the level of serum androgen was significantly associated with the percentage of CD8^+^PD1^+^ T-cells, and a higher serum androgen predicted a worse prognosis in male RCC patients receiving immunotherapy. Based on our findings, we propose a promising treatment regimen, ARi combined with ICIs, which could increase the efficacy of immunotherapy by alleviating exhaustion and enhancing the cytotoxicity of CD8^+^ T-cells.

According to epidemiological surveys, males are more prone to develop cancer in nearly every organ type [34]. However, the influence of lifestyle habits and sociocultural factors cannot be overlooked, as they may contribute to the observed sex bias in certain tumor types [14, 35]. For instance, the higher incidence of smoking among males has been linked to the increased risk of bladder and lung cancer in this population [36]. In contrast, RCC has a constant male-to-female case ratio of 2:1 that is independent of age, year, and region, suggesting that biological factors may play a more significant role in the sex bias of this cancer type. Investigating the underlying mechanisms of sex differences in RCC could provide valuable insights into the biological basis of gender disparities in cancer.

In this study, we found that the malignant cells in male tumors were more aggressive and angiogenic than in female tumors, which was consistent with previous literature [14]. Several studies confirmed the malignant feature of male RCC and suggested that it could be attributed to androgen and AR signaling [15, 37, 38]. AR promotes vasculogenic mimicry formation by modulating lncRNA-TANAR/TWIST1 signals [37]. Additionally, AR-circHIAT1-mediated miR-195-5p/29a-3p/29c-3p/CDC42 signals could promote RCC metastasis [38]. We also found that male tumors had a significantly higher SAA1 expression than the tumor females. Previous studies have reported that SAA1 was significantly associated with poor prognosis in RCC patients [31]. These findings suggest that SAA1 may contribute to sex bias in malignant cells. However, further validation studies are needed to explore the relationship between androgen signaling and SAA1 expression in RCC.

RCC has been widely considered as a typically “hot tumor” with abundant CD8^+^ T-cells infiltrating in TME [39–41]. Our study revealed that the TME of male RCC had a higher level of CD8^+^ T infiltration (“infiltrated type”) than female. Higher CD8^+^ T-cells infiltration was reported to predict a better prognosis in many cancer types due to the cytotoxic function of CD8^+^ T-cells [22, 42, 43]. Unusually, the high infiltration of CD8^+^ T-cells could not predict a better prognosis in RCC patients [33]. Similarly, highly infiltrated CD8^+^ T also didn’t lead to a better prognosis in male RCC. This could be due to specific factors present in the TME of male RCC that impede the typical anticancer effect of CD8^+^ T-cells, and further investigation into these factors may be necessary.

It has been widely recognized that CD8^+^ T-cells could differentiate into distinct subtypes in TME, regular cytotoxic CD8^+^ T-cells play an anticancer role, while exhausted CD8^+^ T-cells were dysfunctional. Several studies involving sex bias revealed the exhausted and dysfunctional CD8^+^ T-cells’ state in bladder [25], prostate [26], and liver cancers [44]. Kwon et al. reported that AR was a direct transcriptional transactivator of Tcf7/TCF1 in progenitor-exhausted CD8^+^ T-cells in bladder cancer [25]. Guan et al. found that AR bound directly to IFNγ, resulting in the dysfunction of CD8^+^ T-cells in prostate cancer [26]. It was also reported that AR could inhibit the activity and stemness of CD8^+^ T-cells by regulating epigenetic and transcriptional differentiation programs [44]. However, the difference in CD8^+^ T-cells between males and females does not exist in all cancer types. The bulk sequencing of lung cancer indicated that the female TME was characterized by significantly higher levels of T-cell dysfunction and inhibitory immune checkpoint factors [24]. Nevertheless, whether the androgen–AR axis plays the same role in RCC has not been evaluated. Our study demonstrated that CD8^+^ T-cells in male RCC were mainly exhausted and dysfunctional compared to female, and confirmed that it was induced by the androgen–AR axis.

The persistence of androgen-induced CD8^+^ T exhaustion may limit the efficacy of ICIs in male RCC, and additional therapeutic strategies were required. ICIs have brought hope to patients with advanced RCC, but the majority of patients do not have durable responses to these agents [45, 46]. It has become clear that highly diverse resistance mechanisms exist in the TME and play major roles in impairing immune responses, leading to resistance to ICIs therapy [47, 48]. A meta-analysis showed that male RCC patients benefited more from immunotherapy [9], which might be explained by abundant infiltrated CD8^+^ T-cells in the TME of male RCC, which can be mobilized after ICIs administration. Nevertheless, because of androgen-induced CD8^+^ T exhaustion, ICIs may not fully activate the infiltrating CD8^+^ T-cells in male RCC. Dong et al. also found that ICIs-based treatment could not reverse the exhaustion of CD8^+^ T-cells in patients with progressive fumarate hydratase-deficient renal cell carcinoma [49]. Therefore, more efficacy drug strategies combined with ICIs for RCC patients are urgently needed.

ARi, which has been reported to inhibit tumor growth and angiogenesis [16, 50], was also a potential drug to improve the efficacy of immunotherapy by reducing androgen-induced CD8^+^ T-cells exhaustion. The application of ARi to RCC has been proposed in several preclinical studies [15, 16, 37, 50]. He et al. reported that ARi could inhibit migration and invasion of RCC by modulating HIF2α/VEGF signals at the level of mRNA and protein expression [16]. The combination of ARi and the receptor tyrosine kinase inhibitor were also considered to overcome drug resistance in RCC [50]. In our study, a combination of ARi and ICIs showed stronger synergistic effects in RCC in vivo, possibly because the androgen-induced immunosuppression in the TME of male RCC was removed by ARi. Additionally, ARi could also reverse higher levels of tumor angiogenesis in male RCC [50]. Altogether, these findings provided novel insights into the combination of immunotherapy with ARi in RCC.

Despite the importance of the results observed in the study, there were some limitations. Firstly, the scRNA-seq data of tumors and adjacent normal kidneys were downloaded from four public datasets. The batch effects between studies cannot be ignored, despite the unification of the analysis process, the harmony R packages were used to eliminate batch effects, and the results were validated by MxIF and FCM in large RCC samples. Secondly, sex chromosome and estrogen may also play an important role in the sex bias of RCC, which was not assessed in this present study. Third, although we offered new insights into the combination of immunotherapy with ARi, clinical trials are necessary to confirm the clinical effectiveness of this approach.

## Perspectives and significance

Our investigation characterized the diversity of the TME in male and female RCC, revealing the presence of highly infiltrated and exhausted CD8^+^ T-cells in male TME. The androgen–AR axis was identified as a factor contributing to the CD8^+^ T-cell exhaustion and dysfunction in male TME. Notably, the combination of ARi with ICIs exhibited a synergistic effect, improving the efficacy of immunotherapy. Ultimately, this study could help medical scientists and clinicians further understand sex bias in RCC, and provide a promising approach for improving the immunotherapy outcomes in male RCC patients.

### Supplementary Information

**Additional file 1:** Method details.

**Additional file 2:** Supplementary figures.

**Additional file 3:** Supplementary tables.

## Data Availability

Publicly available single-cell RNA-seq datasets were downloaded from the Genotypes and phenotypes (phs002065, phs002252), Sequence Read Archive (SRP308561), and European Genome-phenome Archive (EGAS00001002325). All code and other data for validation used in this study could be available from the author Kang Ning (E-mail: ningkang@sysucc.org.cn).

## Abbreviations

AR: Androgen receptor
ARi: Androgen receptor inhibitors
DEGs: Differentially expressed genes
early CD8^+^ Texh: Early exhausted CD8^+^ T-cells
ELISA: Enzyme-linked immunosorbent assay
FCM: Flow cytometry
GrzmB: Granzyme B
GSEA: Gene set enrichment analysis
GSVA: Gene set variation analysis
ICIs: Immune checkpoint inhibitors
IFNγ: Interferon-γ
IHC: Immunohistochemistry
MxIF: Multiplex immunofluorescence
PD1: Programmed cell death protein 1
RCC: Renal cell carcinoma
scRNA-seq: Single-cell RNA sequencing
ssGSEA: Single-sample gene set enrichment analysis
SYSUCC: Sun Yat-sen University Cancer Center
TME: Tumor microenvironment
TNFα: Tumor necrosis factor-α
term CD8^+^ Texh: Terminal exhausted CD8^+^ T-cells
UMAP: Uniform manifold approximation and projection

## References

[1] Sung H, Ferlay J, Siegel RL, Laversanne M, Soerjomataram I, Jemal A (2021). Global cancer statistics 2020: GLOBOCAN Estimates of incidence and mortality worldwide for 36 cancers in 185 countries. CA Cancer J Clin.

[2] Capitanio U, Bensalah K, Bex A, Boorjian SA, Bray F, Coleman J (2019). Epidemiology of renal cell carcinoma. Eur Urol.

[3] Scelo G, Li P, Chanudet E, Muller DC (2018). Variability of sex disparities in cancer incidence over 30 years: the striking case of kidney cancer. Eur Urol Focus.

[4] Aron M, Nguyen MM, Stein RJ, Gill IS (2008). Impact of gender in renal cell carcinoma: an analysis of the SEER database. Eur Urol.

[5] May M, Aziz A, Zigeuner R, Chromecki T, Cindolo L, Schips L (2013). Gender differences in clinicopathological features and survival in surgically treated patients with renal cell carcinoma: an analysis of the multicenter CORONA database. World J Urol.

[6] Lee S, Jeon HG, Kwak C, Kim HH, Byun SS, Lee SE (2012). Gender-specific clinicopathological features and survival in patients with renal cell carcinoma (RCC). BJU Int.

[7] Korman AJ, Garrett-Thomson SC, Lonberg N (2022). The foundations of immune checkpoint blockade and the ipilimumab approval decennial. Nat Rev Drug Discov.

[8] Bi K, He MX, Bakouny Z, Kanodia A, Napolitano S, Wu J (2021). Tumor and immune reprogramming during immunotherapy in advanced renal cell carcinoma. Cancer Cell.

[9] Conforti F, Pala L, Bagnardi V, De Pas T, Martinetti M, Viale G (2018). Cancer immunotherapy efficacy and patients' sex: a systematic review and meta-analysis. Lancet Oncol.

[10] Wang C, Dehghani B, Li Y, Kaler LJ, Proctor T, Vandenbark AA (2009). Membrane estrogen receptor regulates experimental autoimmune encephalomyelitis through up-regulation of programmed death 1. J Immunol.

[11] Polanczyk MJ, Hopke C, Vandenbark AA, Offner H (2007). Treg suppressive activity involves estrogen-dependent expression of programmed death-1 (PD-1). Int Immunol.

[12] Graham J, Abdel-Rahman O, Choueiri TK, Heng DY (2018). Cancer immunotherapy efficacy and patients' sex: a systematic review and meta-analysis. Lancet Oncol 2018;19:737-46: outcomes of metastatic renal cell carcinoma by gender: contrasting results from the International mRCC Database Consortium. Eur Urol.

[13] Dunford A, Weinstock DM, Savova V, Schumacher SE, Cleary JP, Yoda A (2017). Tumor-suppressor genes that escape from X-inactivation contribute to cancer sex bias. Nat Genet.

[14] Peired AJ, Campi R, Angelotti ML, Antonelli G, Conte C, Lazzeri E (2021). Sex and gender differences in kidney cancer: clinical and experimental evidence. Cancers (Basel).

[15] Huang Q, Sun Y, Ma X, Gao Y, Li X, Niu Y (2017). Androgen receptor increases hematogenous metastasis yet decreases lymphatic metastasis of renal cell carcinoma. Nat Commun.

[16] He D, Li L, Zhu G, Liang L, Guan Z, Chang L (2014). ASC-J9 suppresses renal cell carcinoma progression by targeting an androgen receptor-dependent HIF2α/VEGF signaling pathway. Can Res.

[17] Zhai W, Sun Y, Guo C, Hu G, Wang M, Zheng J (2017). LncRNA-SARCC suppresses renal cell carcinoma (RCC) progression via altering the androgen receptor(AR)/miRNA-143-3p signals. Cell Death Differ.

[18] Wang K, Sun Y, Guo C, Liu T, Fei X, Chang C (2019). Androgen receptor regulates ASS1P3/miR-34a-5p/ASS1 signaling to promote renal cell carcinoma cell growth. Cell Death Dis.

[19] Bai JY, Jin B, Ma JB, Liu TJ, Yang C, Chong Y (2021). HOTAIR and androgen receptor synergistically increase GLI2 transcription to promote tumor angiogenesis and cancer stemness in renal cell carcinoma. Cancer Lett.

[20] Gong D, Sun Y, Guo C, Sheu TJ, Zhai W, Zheng J (2011). Androgen receptor decreases renal cell carcinoma bone metastases via suppressing the osteolytic formation through altering a novel circEXOC7 regulatory axis. Clin Transl Med.

[21] Davis AA, Luo J, Zheng T, Dai C, Dong X, Tan L (2023). Genomic complexity predicts resistance to endocrine therapy and CDK4/6 inhibition in hormone receptor-positive (HR+)/HER2-negative metastatic breast cancer. Clin Cancer Res.

[22] Binnewies M, Roberts EW, Kersten K, Chan V, Fearon DF, Merad M (2018). Understanding the tumor immune microenvironment (TIME) for effective therapy. Nat Med.

[23] Klein SL, Flanagan KL (2016). Sex differences in immune responses. Nat Rev Immunol.

[24] Conforti F, Pala L, Pagan E, Bagnardi V, De Pas T, Queirolo P (2021). Sex-based dimorphism of anticancer immune response and molecular mechanisms of immune evasion. Clin Cancer Res.

[25] Kwon H, Schafer JM, Song NJ, Kaneko S, Li A, Xiao T (2022). Androgen conspires with the CD8(+) T cell exhaustion program and contributes to sex bias in cancer. Sci Immunol.

[26] Guan X, Polesso F, Wang C, Sehrawat A, Hawkins RM, Murray SE (2022). Androgen receptor activity in T cells limits checkpoint blockade efficacy. Nature.

[27] Zhang X, Cheng L, Gao C, Chen J, Liao S, Zheng Y (2023). Androgen signaling contributes to sex differences in cancer by inhibiting NF-κB activation in T cells and suppressing anti-tumor immunity. Can Res.

[28] Braun DA, Street K, Burke KP, Cookmeyer DL, Denize T, Pedersen CB (2021). Progressive immune dysfunction with advancing disease stage in renal cell carcinoma. Cancer Cell.

[29] Krishna C, DiNatale RG, Kuo F, Srivastava RM, Vuong L, Chowell D (2021). Single-cell sequencing links multiregional immune landscapes and tissue-resident T cells in ccRCC to tumor topology and therapy efficacy. Cancer Cell.

[30] Young MD, Mitchell TJ, Vieira Braga FA, Tran MGB, Stewart BJ, Ferdinand JR (2018). Single-cell transcriptomes from human kidneys reveal the cellular identity of renal tumors. Science.

[31] Paret C, Schön Z, Szponar A, Kovacs G (2010). Inflammatory protein serum amyloid A1 marks a subset of conventional renal cell carcinomas with fatal outcome. Eur Urol.

[32] Hansen MT, Forst B, Cremers N, Quagliata L, Ambartsumian N, Grum-Schwensen B (2015). A link between inflammation and metastasis: serum amyloid A1 and A3 induce metastasis, and are targets of metastasis-inducing S100A4. Oncogene.

[33] Braun DA, Hou Y, Bakouny Z, Ficial M, Sant' Angelo M, Forman J (2020). Interplay of somatic alterations and immune infiltration modulates response to PD-1 blockade in advanced clear cell renal cell carcinoma. Nat Med.

[34] Dart A (2020). Sexual dimorphism in cancer. Nat Rev Cancer.

[35] Sun Q, Yu D, Fan J, Yu C, Guo Y, Pei P (2022). Healthy lifestyle and life expectancy at age 30 years in the Chinese population: an observational study. Lancet Public Health.

[36] Lortet-Tieulent J, Goding Sauer A, Siegel RL, Miller KD, Islami F, Fedewa SA (2016). State-level cancer mortality attributable to cigarette smoking in the United States. JAMA Intern Med.

[37] You B, Sun Y, Luo J, Wang K, Liu Q, Fang R (2021). Androgen receptor promotes renal cell carcinoma (RCC) vasculogenic mimicry (VM) via altering TWIST1 nonsense-mediated decay through lncRNA-TANAR. Oncogene.

[38] Wang K, Sun Y, Tao W, Fei X, Chang C (2017). Androgen receptor (AR) promotes clear cell renal cell carcinoma (ccRCC) migration and invasion via altering the circHIAT1/miR-195-5p/29a-3p/29c-3p/CDC42 signals. Cancer Lett.

[39] Zhang Y, Narayanan SP, Mannan R, Raskind G, Wang X, Vats P (2021). Single-cell analyses of renal cell cancers reveal insights into tumor microenvironment, cell of origin, and therapy response. Proc Natl Acad Sci USA.

[40] Motzer RJ, Robbins PB, Powles T, Albiges L, Haanen JB, Larkin J (2020). Avelumab plus axitinib versus sunitinib in advanced renal cell carcinoma: biomarker analysis of the phase 3 JAVELIN Renal 101 trial. Nat Med.

[41] Gerlinger M, Horswell S, Larkin J, Rowan AJ, Salm MP, Varela I (2014). Genomic architecture and evolution of clear cell renal cell carcinomas defined by multiregion sequencing. Nat Genet.

[42] Sasson SC, Slevin SM, Cheung VTF, Nassiri I, Olsson-Brown A, Fryer E (2021). Interferon-gamma-producing CD8(+) tissue resident memory T cells are a targetable hallmark of immune checkpoint inhibitor-colitis. Gastroenterology.

[43] Wang T, Shen Y, Luyten S, Yang Y, Jiang X (2020). Tissue-resident memory CD8(+) T cells in cancer immunology and immunotherapy. Pharmacol Res.

[44] Yang C, Jin J, Yang Y, Sun H, Wu L, Shen M (2022). Androgen receptor-mediated CD8(+) T cell stemness programs drive sex differences in antitumor immunity. Immunity.

[45] Peng YL, Xiong LB, Zhou ZH, Ning K, Li Z, Wu ZS (2022). Single-cell transcriptomics reveals a low CD8(+) T cell infiltrating state mediated by fibroblasts in recurrent renal cell carcinoma. J Immunother Cancer.

[46] Antonia SJ, Borghaei H, Ramalingam SS, Horn L, De Castro CJ, Pluzanski A (2019). Four-year survival with nivolumab in patients with previously treated advanced non-small-cell lung cancer: a pooled analysis. Lancet Oncol.

[47] Vesely MD, Zhang T, Chen L (2022). Resistance mechanisms to anti-PD cancer immunotherapy. Annu Rev Immunol.

[48] Bagchi S, Yuan R, Engleman EG (2021). Immune checkpoint inhibitors for the treatment of cancer: clinical impact and mechanisms of response and resistance. Annu Rev Pathol.

[49] Dong P, Zhang X, Peng Y, Zhang Y, Liu R, Li Y (2022). Genomic characteristics and single-cell profiles after immunotherapy in fumarate hydratase-deficient renal cell carcinoma. Clin Cancer Res.

[50] Adelaiye-Ogala R, Damayanti NP, Orillion AR, Arisa S, Chintala S, Titus MA (2018). Therapeutic targeting of sunitinib-induced AR phosphorylation in renal cell carcinoma. Can Res.

