# Supplementary material for: Sex differences in renal cell carcinoma: a single-cell analysis reveals exhausted CD8+ T-cells highly infiltrated in males
Source: Biol Sex Differ. 2023 Sep 15;14:58. doi: 10.1186/s13293-023-00540-9 (PMC10503187; doi:10.1186/s13293-023-00540-9)
Supplement: Supplementary file 1 — Additional file 1: Method details. [file 13293_2023_540_MOESM1_ESM.pdf]

Additional file 1

1. Method

1.1 Clinical data and meta-analysis

After approval by the Institutional Review Board, the data of 1102 RCC patients who underwent radical nephrectomy between January 1, 2000, and December 31, 2020, were analyzed for gender differences. Overall survival was defined as the time from the date of radical nephrectomy to the date of death due to any cause or censored at the date of the last follow-up. As previously described(1), a meta-analysis was conducted on immunotherapy for metastatic RCC. Lastly, the data from five randomized controlled trials were included(2-6), and the main characteristics and results of these studies were summarized in Supplementary Table 1A and Supplementary Figure 1A.

1.2 Single-cell data acquisition and screening criteria

Publicly available single-cell RNA-seq datasets were downloaded from the Genotypes and phenotypes (phs002065, phs002252), Sequence Read Archive (SRP308561) and European Genome-phenome Archive (EGAS00001002325)(7-10). Datasets included in this study had to meet the following criteria: (1) involved primary RCC; (2) provided unnormalized count data; (3) were generated using the 10x Genomics Chromium platform; (4) came from untreated RCC patients. Sex-related genes (XIST, RPS4Y1) were used to identify sex groups in each sample (Supplementary Figure 1C). Finally, we obtained 165,326 cells from 19 patients (19 tumors and 17 adjacent normal kidney tissues) from 4 datasets (Supplementary Figure 1B and Supplementary Table 1B).

1.3 Single-cell data quality control and subcluster detection

To unify the upstream analysis process of each dataset, scRNA-seq fastq files were downloaded and processed using Cell Ranger v5.0.1 (10x genomics). Raw gene expression matrices were imported and processed using the Seurat R package (version 4.1.0). Low-quality cells were removed the following 4 measurements: 1) cells that had either fewer than 200 or over 5000 unique molecular identifiers (UMIs), over 20,000 or less than 300 expressed genes or over 15% UMIs derived from the mitochondrial genome, or over 2.5% UMIs derived from the erythrocytic genome; 2) cells with an

average expression level of less than 2 for a curated list of housekeeping genes; 3) cells co-expressing EPCAM and PTPRC. 4) Doublets were detected by DoubletFinder R package (version 2.0.3) for a single sample and manually detected the doublets in re-clustering the cell types.

Dissociation-induced genes were regressed to avoid effects on the clustering, and batch effects were minimized (lambda = 1.5) using the Harmony R package (version 1.0) (11). After normalized to the total cellular UMI counts, the gene expression matrices were scaled (scale. factor = 10,000) by regressing the total cellular UMI counts and percentage of mitochondrial genes. Highly variable genes (top 3000) were extracted using the Seurat "FindVariableGenes" function. Then, we performed principal component analysis (PCA) analysis using high variable genes, and significant PCs (top 20) were selected to perform dimension reduction. Clusters were found using the "FindClusters" function (dims.use = 1:20, resolution = 0.5). The Uniform Manifold Approximation and Projection (UMAP) analysis was used for dimension. We defined 8 major lineages epithelial cell, endothelial cell, myeloid cell, fibroblast, T cell, B cell, NK cell, and proliferating cell. Then, after re-clustering epithelial cells, T-cells and myeloid cells, several subclusters were produced for biological differences analysis.

1.4 Differential gene expression analysis and gene set enrichment analysis

We used the "FindMarkers" function to derive differentially expressed genes (DEGs) from each cluster, and genes with a p-value < 0.01 and average log2 fold change ≥0.25 were retained (Table S2A, S3A and S4A). Gene set enrichment analysis (GSEA) was performed to analyze different pathways in different subclusters on some gene signatures(12). Gene set variation analysis (GSVA) was also performed to estimate pathway activity scores for single cells(13). Then, the differential activities of pathways were calculated using the limma R package (version 3.46.0)(14). All gene signatures used in this study are shown in Supplementary Table S2B, S3B and S4B. Single-sample gene set enrichment analysis (ssGSEA) was conducted to calculate the androgen receptor activity score and T-cell exhaustion score for each cell(15).

1.5 Pseudotime trajectory analysis

The cell lineage trajectory of $CD8^+$ T cell and macrophage was inferred using the Monocle2 R package(16). We used the ''FindMarkers'' function to identify DEGs from each cluster and genes

with a p-value < 0.01 were used to order the cells in pseudotime analysis. We used the average expression (measured by log2 (TPM + 1) of exhaustion- and dysfunction-related genes to define the exhaustion and pro-dysfunction score for $CD8^+$ T-cells. We analyzed the exhaustion and dysfunction scores along with pseudotime in CD8+ T-cells and anti-inflammatory and pro-inflammatory scores along with pseudotime in macrophages.

1.6 Multiplex immunofluorescence (MxIF) assay and image analysis

Formalin-fixed paraffin-embedded (FFPE) slides of sixty patients were collected for MxIF to assess the infiltration of $CD8^+$ T-cells. The FFPE slides were sequentially stained with antibodies specific for CD3 (CD3 cells, ab16669, Abcam), CD8 (T-cells, ab217344, Abcam), PD1 (ab237728, Abcam), and pan-cytokeratin (tumor cells, ab7753, Abcam). The nuclei were highlighted using 4′,6-diamidino-2-phenylindole (DAPI). Image analysis was performed using the HALO software (version 3.3; Indicalab, New Mexico, USA), including tissue segmentation, cell segmentation and phenotyping to assign each cell to a phenotypic category (Supplementary Table S3C).

The CD3 immunofluorescence slides were scanned at ×10 magnification, and we marked the boundary between tumor and stroma using the HALO software. According to the spatial location of CD3 positive cells and tumor cells, patients were divided into three groups: infiltrated, excluded and desert. The number of $CD3^+$ cells was calculated by the average of 10 representative fields of view at a magnification of 200×. A tumor was categorized as "desert" when its average $CD3^+$ cells were lower than 10 cells in an area of the tumor and peritumor region. A tumor was categorized as "infiltrated" when its average $CD3^+$ cells were greater than 10 cells in an area of the tumor and peritumor region. A tumor was categorized as "excluded" when its average $CD3^+$ cells were lower than 10 cells only in an area of the tumor(17).

1.7 Flow cytometry (FCM)

Human primary RCC tissues were obtained from the operating room during nephrectomy. After rinsing in HBSS (Gibco) and cut into small pieces, tissues were digested for 30 min at 37°C with agitation in a digestion solution containing 25μg/ml Liberase TM (Roche) and 50μg/ml DNase (Sigma) in RPMI (Gibco). At the same time, mouse tumors were mechanically disrupted using the Tumor Cell Isolation Kit (Miltenyi, Cat: 130110187) according to the manufacturer's instructions.

Tissue dissociation fluid was passed through 70 μm filters to prepare single-cell suspensions, and excess red blood cells were removed using Red Blood Cell Lysis Buffer. For cytokine production experiments, the cells were re-stimulated by the Leukocyte Activation Cocktail kit (BD Pharmingen, 550583) for 8 hours at 37°C. After being washed twice with PBS, single cells were stained at 4°C with APC-CY7 fixable viability dye (BD Pharmingen, Cat:565388) for 15 min. FcR block was incubated with the samples for 20 min, and extracellular surface markers were subsequently added. All intracellular staining was performed with the Cytofix/Cytoperm Soln Kit (BD Pharmingen, 554714). All samples were acquired on a cytoFLEX flow cytometer and analyzed on FlowJo (version 10.8.1). The fluorochrome-conjugated antibodies included in the T cell exhaustion panel are listed in Supplementary Table S3D.

1.8 Human $CD8^+$ T-cells culture

Peripheral blood mononuclear cells (PBMC) from healthy donors were first isolated from a buffy coat by density gradient centrifugation using Lymphocyte Separation Medium (Biosharp). $CD8^+$ T-cells were isolated from PBMC using EasySep Mouse $CD8^+$ T Cell Isolation Kit (Stemcell Technologies). Subsequent experiments were carried out after confirming $CD8^+$ T cell population was >95% pure. For all the experiments, $CD8^+$ T-cells were cultivated with the X-VIVO medium (LONZA) supplemented with 10% fetal bovine serum (FBS) at the density of 1.5×10^6 cells per ml per cm^2 with additional interleukin-2 supplement (100 IU/mL, Miltenyi Biotec.) and ImmunoCult™ Human CD3/CD28 T Cell Activator (10μl/mL, InvivoGen).

1.9 CD8+ T cell cytotoxicity assay

For the cytotoxicity study, lymph nodes from OT-I mice were mechanically dissociated, and OT-I $CD8^+$ T-cells were isolated using the EasySep Mouse $CD8^+$ T Cell Isolation Kit (Stemcell Technologies). OT-I $CD8^+$ T-cells were cultured in complete RPMI medium [containing 10% FBS + 1× GlutaMax (Gibco), 100 U/mL penicillin-streptomycin, 1 mM sodium pyruvate and 50 μmol/L 2-mercaptoethanol]. OT-I $CD8^+$ T-cells were stimulated with SIINFEKL (10 ng/mL) and IL-2 (10 ng/mL) for 5 days to generate cytotoxic T-cells. Renca cells were transfected with a lentiviral vector (LV)-mCherry-OVA and fluorescence-activated cell sorting was used to select stable Renca-mCherry-OVA cell lines. OT-I $CD8^+$ T-cells were co-cultured with Renca-mCherry-OVA at a ratio of

1:1, with or without dihydrotestosterone (DHT, CATO Research Chemicals Inc.) for 8 hours. The co-cultured cells were evaluated with a crystal violet staining solution, and the cells were counted using ImageJ (version 1.8.0). FCM of 7-AAD was also used to evaluate the apoptosis of Renca-mCherry-OVA.

1.10 Immunohistochemistry (IHC) and Enzyme-linked immunosorbent assay (ELISA)

We collected tissue-embedded wax and serum from RCC patients receiving immunotherapy at our hospital for IHC and ELISA. After deparaffinization, slides were hydrated in alcohol and endogenous peroxidase activity was quenched for 30 min in 10% hydrogen peroxide. Antigen epitope retrieval was induced by microwave heating. To examine the expression pattern of candidate antibodies in RCC tissues, the sections were immunostained with primary antibodies for 1 hour at 37°C. Then, enzyme-labeled goat anti-mouse/rabbit IgG polymer (Cat: PV-6000, ZSGB-BIO, Beijing) was added and incubated at room temperature for 20 minutes. The signal was detected using DAB (ZSGB-BIO, Beijing) following the manufacturer's protocol. The IHC score of Gal1 was calculated by the HALO software (Indicalab, New Mexico, USA). The antibodies used were: PD1 (human, ab52587, Abcam); CD8 (human, ab217344, Abcam); PD1 (mouse, ab214421, Abcam); GrzmB (mouse,46890S, CST); IFNγ (mouse, DF6045, Affinity Biosciences); and TNFα (mouse, ab34674, Abcam). All serums were stored in Bio-bank after collection at our hospital. Plasma testosterone concentration was determined using a testosterone ELISA kit (RUIXIN biotech, Cat: RXJ105886H) according to the manufacturer's recommendations.

1.11 *In vivo* experiments

BALB/c mice were purchased from Vital River (Beijing, China) and were maintained in specific pathogen-free conditions in the animal laboratory at our hospital. The murine cell line of renal cancer (Renca) was kindly provided by Professor Luo (Center for Precision Medicine, Sun Yat-sen University, Guangzhou, China). Renca were cultured in a complete RPMI medium [containing 10% FBS, 0.1mM NEAA, 100 U/mL penicillin-streptomycin, 1 mM sodium pyruvate, and 2 mM L-glutamine]. In the subcutaneous tumorigenesis, Renca (1×10^6 cells) resuspended in 100 μL PBS were injected into the right side of the skin of 4 weeks male or female BALB/c mice. Tumor surface

area (0.52 × Length × Width^2) was measured using an electronic caliper starting 8 days post-implantation.

To explore the effect of androgen on tumor growth, BALB/c mice with subcutaneous tumors were divided into four groups: male (normal), female (normal), male (castration), and female (androgen). Besides, male BALB/c mice with subcutaneous tumors were divided into four other groups to explore the effects of androgen receptor inhibitors (ARi) on tumor growth: control (PBS), ENZ (enzalutamide), anti-PD1, and ENZ+anti-PD1. For the castration group, male mice were anesthetized with isoflurane and placed on a surgical pad. A 1-2 mm surgical incision was made below the scrotum. After the left and right testis were exposed, the spermatic cord was ligated, and the testis was removed. For the androgen group, slow-releasing pellets with DHT (1.5 mg per mouse) were injected into female mice at 5 weeks of age. For enzalutamide experiments, mice were treated daily with either vehicle control or enzalutamide (Beyotime, SC0074) through oral gavage (25 mg/kg) starting on 8 days post Renca subcutaneous injection. Anti-PD1 (clone RMP1-14, BioXCell) was injected i.p. (200μg/mouse) every 2 days.

1.12 Statistical analysis

The data were analyzed using the GraphPad Prism (version 8; San Diego, California, US) and R (version 4.1.0; Auckland, New Zealand) software. Comparisons between males and females were performed using the unpaired paired Wilcoxon test. We constructed survival curves using the Kaplan-Meier method, and their equality was compared via the log-rank test. The tumor growth in mice was measured using the method of repeated-measures ANOVA. All data are presented as means ± standard deviations (SDs). * $p < 0.05$, ** $p < 0.01$, *** $p < 0.001$. Statistical significance was set at p-values less than 0.05.

2. References

1. Conforti F, Pala L, Bagnardi V, De Pas T, Martinetti M, Viale G*, et al.* Cancer immunotherapy efficacy and patients' sex: a systematic review and meta-analysis. *Lancet Oncol* 2018;**19**(6):737-46 doi 10.1016/s1470-2045(18)30261-4.
2. Motzer RJ, Escudier B, McDermott DF, George S, Hammers HJ, Srinivas S*, et al.* Nivolumab versus Everolimus in Advanced Renal-Cell Carcinoma. *N Engl J Med* 2015;**373**(19):1803-13 doi 10.1056/NEJMoa1510665.
3. Motzer RJ, Penkov K, Haanen J, Rini B, Albiges L, Campbell MT*, et al.* Avelumab plus Axitinib versus Sunitinib for Advanced Renal-Cell Carcinoma. *N Engl J Med* 2019;**380**(12):1103-15 doi 10.1056/NEJMoa1816047.
4. Motzer R, Alekseev B, Rha SY, Porta C, Eto M, Powles T*, et al.* Lenvatinib plus Pembrolizumab or Everolimus for Advanced Renal Cell Carcinoma. *N Engl J Med* 2021;**384**(14):1289-300 doi 10.1056/NEJMoa2035716.
5. Rini BI, Plimack ER, Stus V, Gafanov R, Hawkins R, Nosov D*, et al.* Pembrolizumab plus Axitinib versus Sunitinib for Advanced Renal-Cell Carcinoma. *N Engl J Med* 2019;**380**(12):1116-27 doi 10.1056/NEJMoa1816714.
6. Choueiri TK, Motzer RJ, Rini BI, Haanen J, Campbell MT, Venugopal B*, et al.* Updated efficacy results from the JAVELIN Renal 101 trial: first-line avelumab plus axitinib versus sunitinib in patients with advanced renal cell carcinoma. *Ann Oncol* 2020;**31**(8):1030-9 doi 10.1016/j.annonc.2020.04.010.
7. Braun DA, Street K, Burke KP, Cookmeyer DL, Denize T, Pedersen CB*, et al.* Progressive immune dysfunction with advancing disease stage in renal cell carcinoma. *Cancer Cell* 2021;**39**(5):632-48.e8 doi 10.1016/j.ccell.2021.02.013.
8. Krishna C, DiNatale RG, Kuo F, Srivastava RM, Vuong L, Chowell D*, et al.* Single-cell sequencing links multiregional immune landscapes and tissue-resident T cells in ccRCC to tumor topology and therapy efficacy. *Cancer Cell* 2021;**39**(5):662-77.e6 doi 10.1016/j.ccell.2021.03.007.
9. Bi K, He MX, Bakouny Z, Kanodia A, Napolitano S, Wu J*, et al.* Tumor and immune reprogramming during immunotherapy in advanced renal cell carcinoma. *Cancer Cell* 2021;**39**(5):649-61.e5 doi 10.1016/j.ccell.2021.02.015.
10. Young MD, Mitchell TJ, Vieira Braga FA, Tran MGB, Stewart BJ, Ferdinand JR*, et al.* Single-cell transcriptomes from human kidneys reveal the cellular identity of renal tumors. *Science* 2018;**361**(6402):594-9 doi 10.1126/science.aat1699.
11. van den Brink SC, Sage F, Vértesy Á, Spanjaard B, Peterson-Maduro J, Baron CS*, et al.* Single-cell sequencing reveals dissociation-induced gene expression in tissue subpopulations. *Nat Methods* 2017;**14**(10):935-6 doi 10.1038/nmeth.4437.
12. Subramanian A, Tamayo P, Mootha VK, Mukherjee S, Ebert BL, Gillette MA*, et al.* Gene set enrichment analysis: a knowledge-based approach for interpreting genome-wide expression profiles. *Proc Natl Acad Sci U S A* 2005;**102**(43):15545-50 doi 10.1073/pnas.0506580102.
13. Hänzelmann S, Castelo R, Guinney J. GSVA: gene set variation analysis for microarray and RNA-seq data. *BMC Bioinformatics* 2013;**14**:7 doi 10.1186/1471-2105-14-7.
14. Ritchie ME, Phipson B, Wu D, Hu Y, Law CW, Shi W*, et al.* limma powers differential expression analyses for RNA-sequencing and microarray studies. *Nucleic Acids Res* 2015;**43**(7):e47 doi 10.1093/nar/gkv007.
15. Barbie DA, Tamayo P, Boehm JS, Kim SY, Moody SE, Dunn IF*, et al.* Systematic RNA interference reveals that oncogenic KRAS-driven cancers require TBK1. *Nature* 2009;**462**(7269):108-12 doi 10.1038/nature08460.
16. Qiu X, Mao Q, Tang Y, Wang L, Chawla R, Pliner HA*, et al.* Reversed graph embedding resolves complex single-cell trajectories. *Nat Methods* 2017;**14**(10):979-82 doi 10.1038/nmeth.4402.
17. Mariathasan S, Turley SJ, Nickles D, Castiglioni A, Yuen K, Wang Y*, et al.* TGFβ attenuates tumour response to PD-L1 blockade by contributing to exclusion of T cells. *Nature* 2018;**554**(7693):544-8 doi 10.1038/nature25501.
