# Supplementary material for: Sex differences in renal cell carcinoma: a single-cell analysis reveals exhausted CD8+ T-cells highly infiltrated in males
Source: Biol Sex Differ. 2023 Sep 15;14:58. doi: 10.1186/s13293-023-00540-9 (PMC10503187; doi:10.1186/s13293-023-00540-9)
Supplement: Supplementary file 2 — Additional file 2: Supplementary figures. [file 13293_2023_540_MOESM2_ESM.pdf]

Figure S1

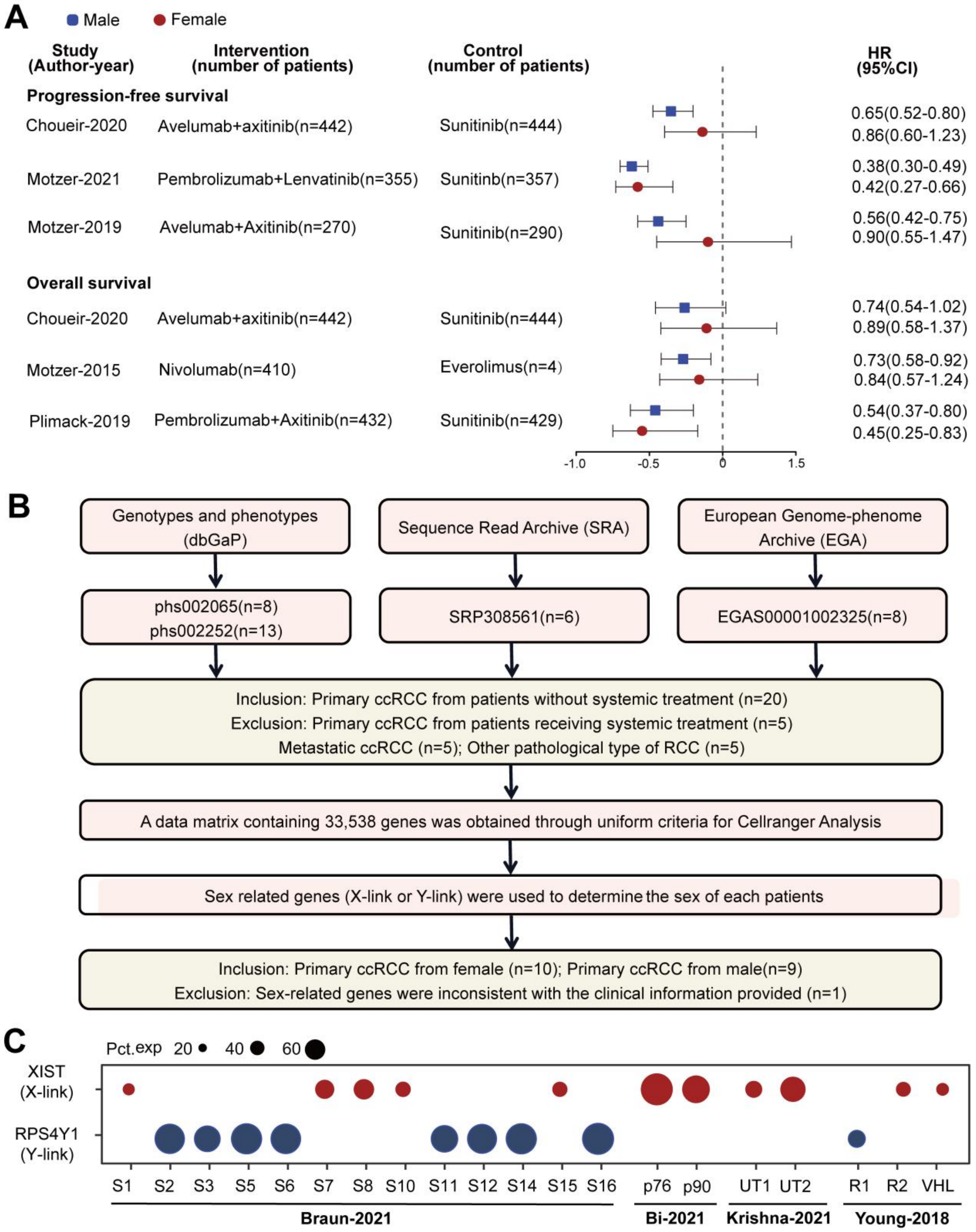

Figure S1. Meta-analysis of RCTs and flow diagram of scRNA-seq data screening, related to Figure 1

(A) Hazard ratios of OS and PFS for patients assigned to immunotherapy, compared with those assigned to control treatment, by sex from various immunotherpy RCTs .

(B) The flow diagram of single-cell sequencing data screening from four studies.

(C) The dot plot showing the X-link or Y-link gene in primary RCC samples after screening.

OS: overall survival; **PFS**: progression-free survival; **RCC**: renal cell carcinoma; **RCTs**: randomized controlled trials; **scRNA-seq**: single-cell sequencing

Figure S2

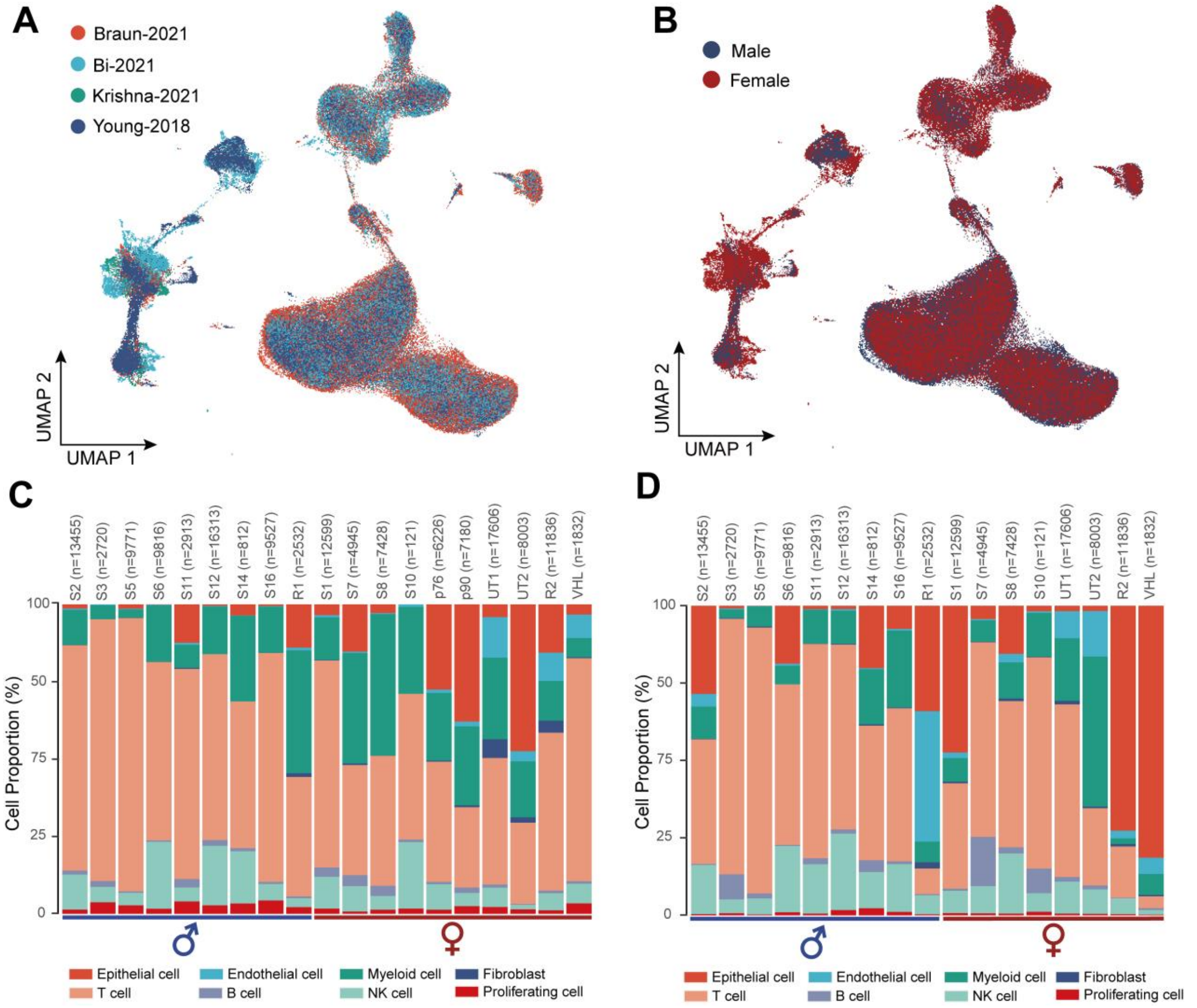

Figure S2. Individual cell subsets identified through scRNA-seq in different studies, genders and samples, related to Figure 1.

(A, B) The UMAP plot showing studies origin and genders origin.

(C, D) Histogram illustrating the percentage of cell types in each tumor sample and adjacent normal tissue sample.

scRNA-seq:single-cell RNA sequencing; **UMAP**: uniform manifold approximation and projection

Figure S3

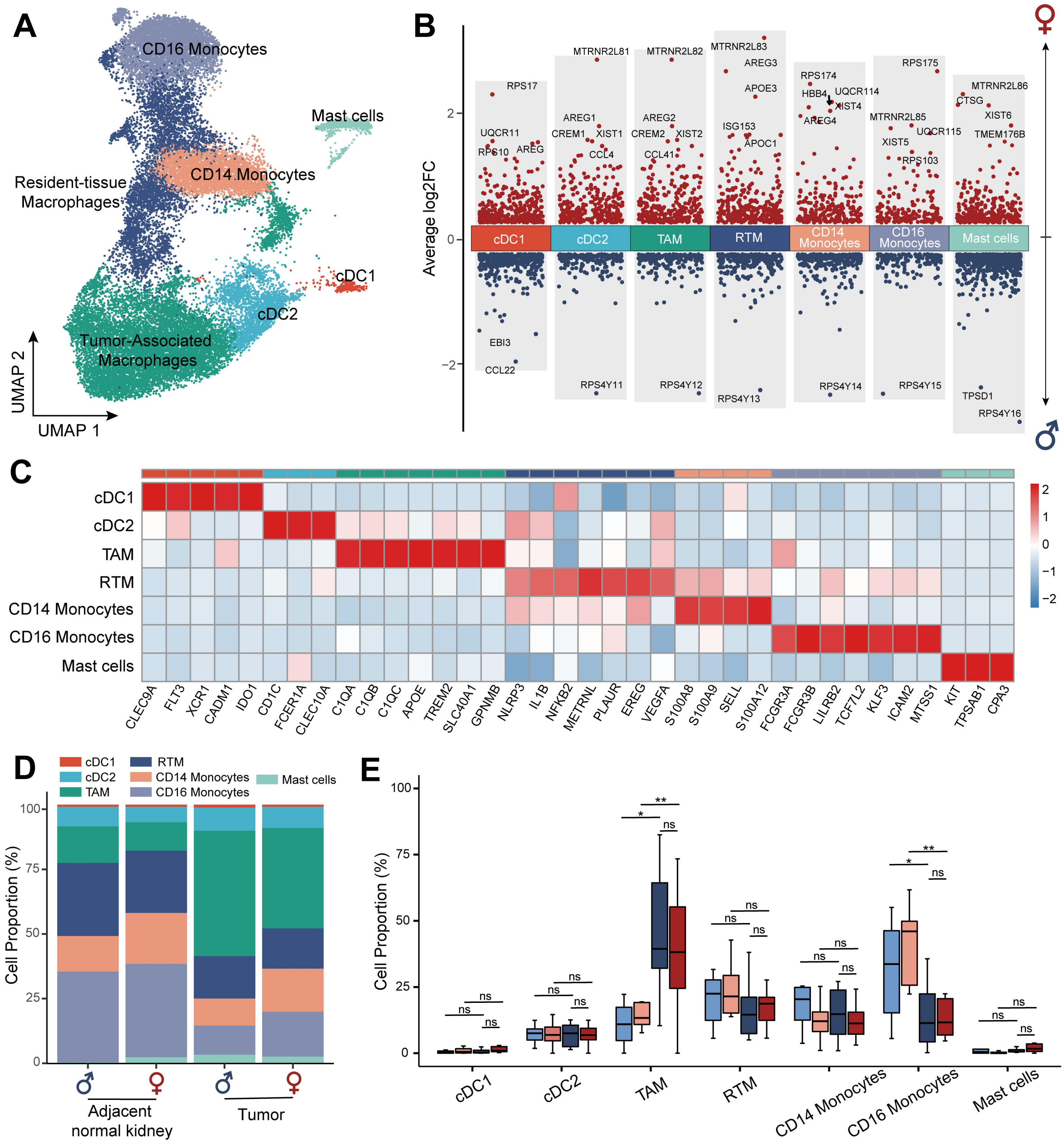

Figure S3 . Identification and characterization of infiltrating myeloid cell in male and female.

(A) The UMAP plot showing different myeloid cells subtypes, colored and labeled by cell type.

(B) The volcano plot shows DEGs between male (blue dots) and female (red dots) in different myeloid cells subsets.

(C) Heatmap showing the expression of marker genes in different myeloid cells subtypes.

(D, F) Histogram and box plots illustrating the percentage of myeloid cells subtypes in different groups.

DEGs: differential gene expression; **UMAP**:uniform manifold approximation and projection.

Figure S4

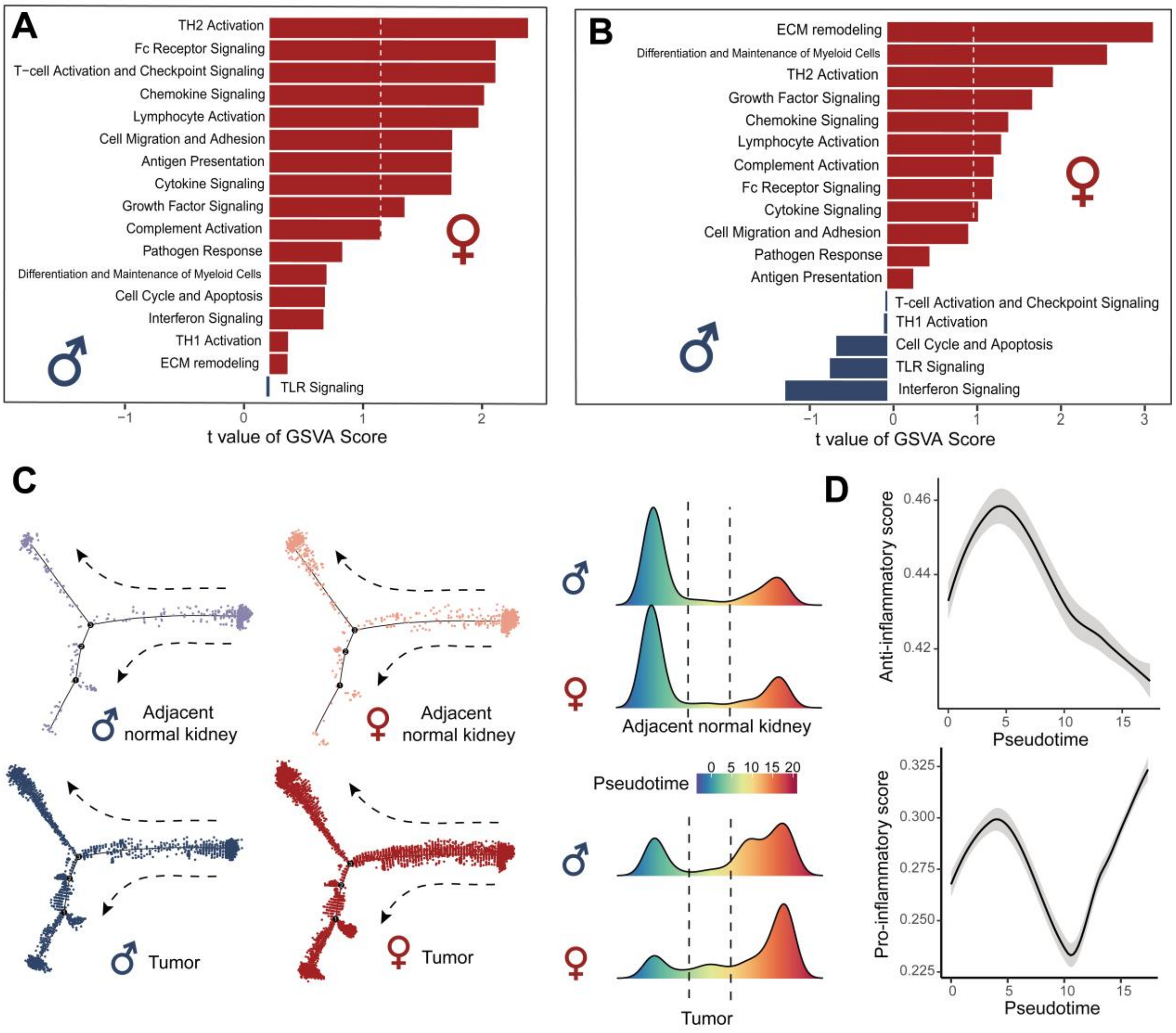

Figure S4. Analysis of the function and transition states of infiltrating macrophage in male and female samples.

(A, B) Differentially enriched pathways were scored per cell by GSVA in infiltrating macrophage between male and female adjacent normal kidney and tumor tissue.

(C) Pseudotime-ordered analysis and density-distribution map of infiltrating macrophage from male and female samples.

(D) Two-dimensional plots showing the change of expression scores for genes related to anti-inflammatory and pro-inflammatory.

GSVA: gene set variation analysis

Figure S5

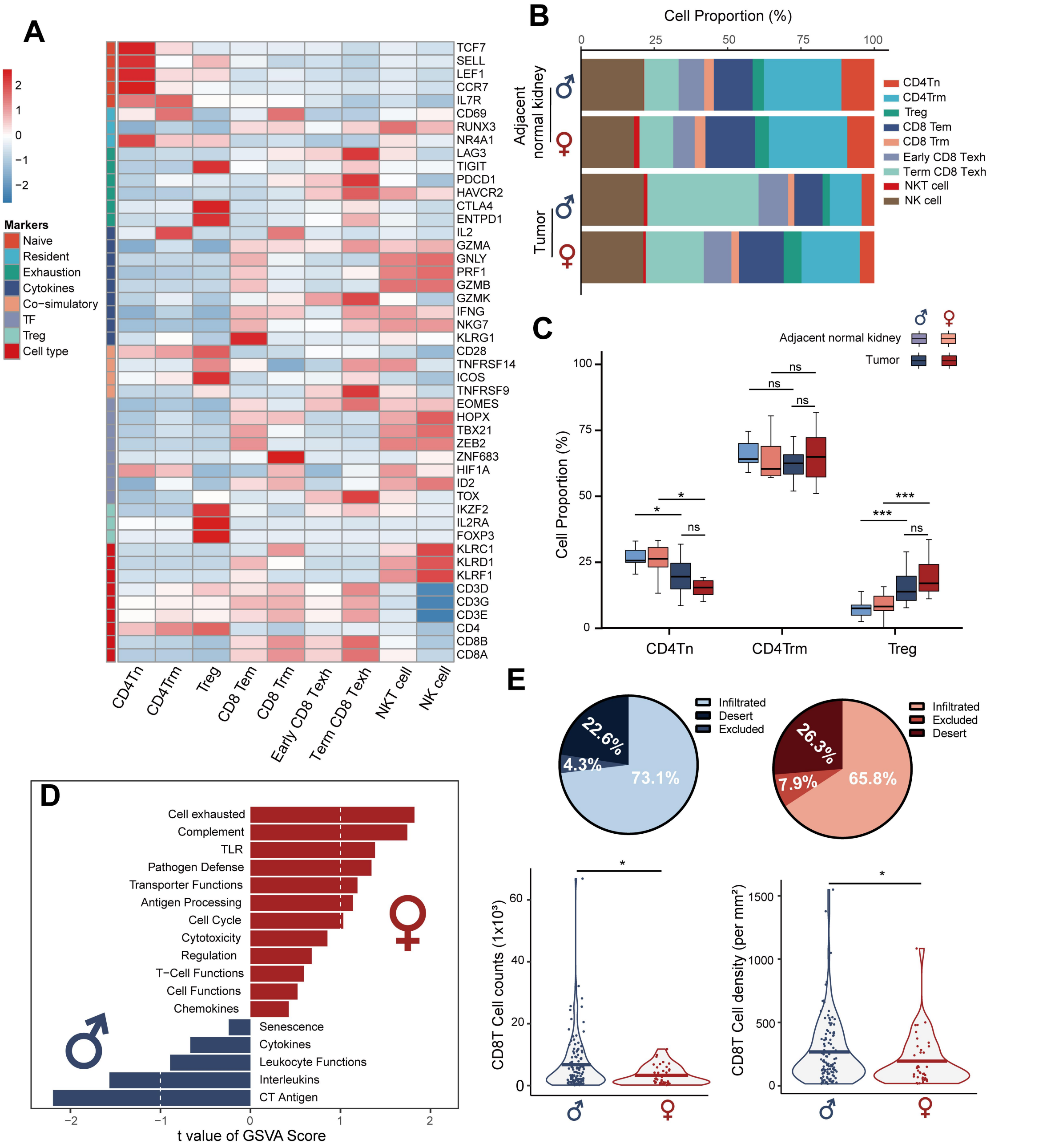

Figure S5 . Identification and characterization of infiltrating T cell in male and female, related to Figure 3.

(A) Heatmap of cell lineage and functional markers providing phenotypic information for individual T cell subtypes.

(B) Histogram showing the percentage of individual T cell subtypes in different groups.

(C) Boxplots illustrating the percentage of infiltrating $CD4^{+}$ T cell subtypes in tumor and adjacent normal kidney of males and females.

(D) Differentially expressed pathways were scored per cell by GSVA in infiltrating $CD8^{+}$ T cell between male and female adjacent normal kidney tissue.

(E) The MxIF data published by others (Braun et al., DOI:10.1038/s41591-020-0839-y) were analyzed by gender grouping.

GSVA: gene set variation analysis; **MxIF**: multiplex immunofluorescence

Figure S6

A

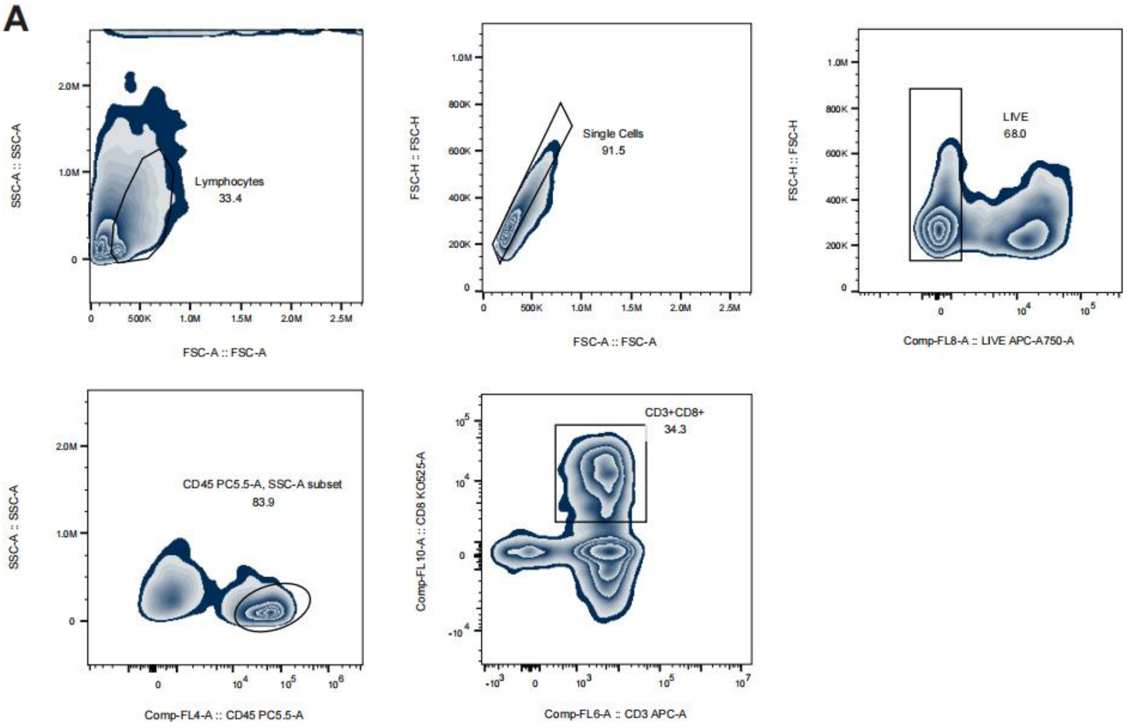

Figure S6 . Gating strategy for CD8+ T cells FCM, related to Figure 4.

A. Tumor-infiltrating CD8+ T cells isolating from RCC

B. Tumor-infiltrating CD8+ T cells isolating from mouse tumors.

FCM: flow cytometry; **RCC**: renal cell carcinoma

Figure S7

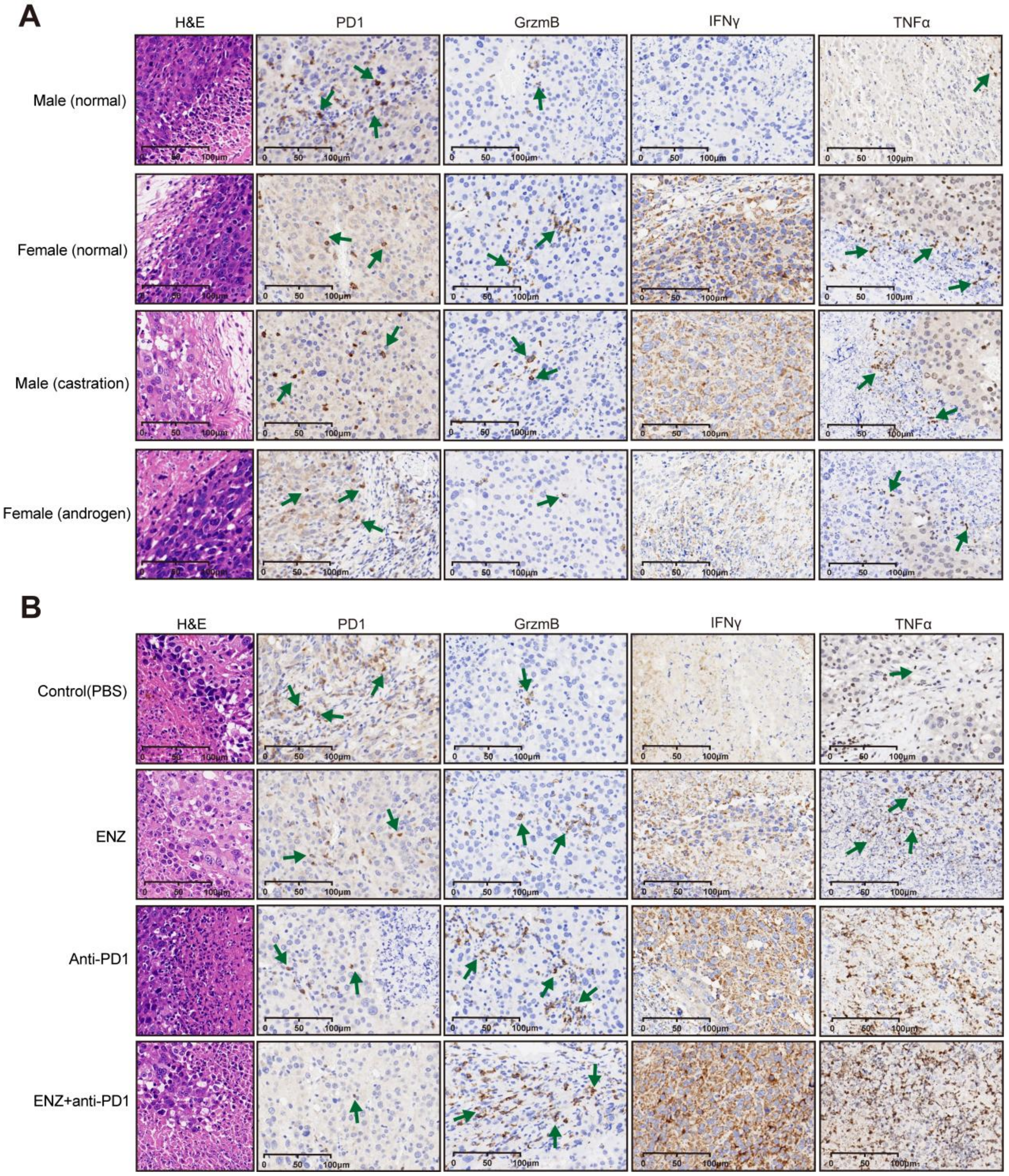

Figure S7 . The expression of cytotoxicity and exhaustion markers of CD8+ T-cells, related to Figure 6.

A. IHC was used to evaluate the expression of cytotoxicity and exhaustion markers of CD8+ T-cells in androgen experiments.

B. IHC was used to evaluate the expression of cytotoxicity and exhaustion markers of CD8+ T-cells in immunotherapy experiments.

IHC: immunohistochemistry
